# DeepBBB: A Data-Composition-Aware Graph Screening Workflow for BBB-Focused CNS Library Construction and Prospective PAMPA-BBB Evaluation

**DOI:** 10.3390/ph19081319

**Published:** 2026-08-21

**Authors:** Ziying Xu, Wei Xia, Haiqiang Wu, Haiping Zhang

**Affiliations:** 1 School of Pharmacy, Shenzhen University Medical School, Shenzhen University, Shenzhen 518055, China; xu_balabala@126.com; 2 Department of Chemistry, New York University, New York, NY 10003, USA; wx2237@nyu.edu; 3 Faculty of Pharmaceutical Sciences, Shenzhen University of Advanced Technology, Shenzhen 518055, China

**Keywords:** blood–brain barrier, PAMPA-BBB, graph neural networks, CNS drug discovery, class imbalance, virtual screening, BBB-focused library

## Abstract

**Background/Objectives:** Blood–brain barrier (BBB) permeability is a major practical obstacle in central nervous system (CNS) drug discovery, because only a small fraction of drug-like molecules achieve sufficient brain exposure. **Methods:** We present DeepBBB, a graph-based, data-composition-aware screening workflow for predicting BBB permeability and for constructing BBB-focused screening libraries from commercial chemical space. Rather than introducing a new graph-learning architecture, the workflow combines standard graph convolutional and graph-transformer models with deliberate control of training-set composition, commercial-library filtering, chemical-space profiling, and prospective experimental evaluation. Three model variants were trained on the Blood–Brain Barrier Database (B3DB): a baseline classifier/regressor pair (DeepBBB_V1_BC/RG), a variant trained with a more strongly negative-enriched configuration (DeepBBB_V2_BC), and a graph-transformer counterpart (DeepBBB_trans_BC/RG). Because the sample-level split assignments and per-compound predictions from the original runs were not recoverable, the archived summary metrics are reported descriptively in the main text and are not used to support calibration, scaffold-level validity, or generalization. Applying the workflow to the ChemDiv collection (~1.5 million compounds) and the Enamine REAL lead-like space (~1.7 billion compounds) produced three progressively more stringently filtered BBB-focused libraries (21,991; 4,808,885; and 151,790 compounds). **Results:** Analysis of available processed data indicated that the predicted BBB-permeable set occupies a compact, BBB-compatible property region. Physicochemical, fragment, and scaffold summaries were interpreted descriptively at the constructed-library level. In a first prospective campaign, one of 12 tested candidate compounds was PAMPA-BBB-positive (all-tested molecular-level positive fraction 8.3%; exact 95% CI 0.2–38.5%). In a second campaign, five of 35 tested candidate compounds were PAMPA-BBB-positive (14.3%; exact 95% CI 4.8–30.3%); 13 compounds were not quantifiable and were not treated as ordinary CNS-negative measurements, and the two campaigns differed in compound source, selection strategy, and assay setting, so the numerical difference is reported descriptively rather than causally. **Conclusions:** Together, these results support the feasibility of BBB-focused computational filtering and a PAMPA-BBB evaluation workflow for CNS-oriented discovery.

## 1. Introduction

Diseases of the central nervous system (CNS), including neurodegenerative disorders, psychiatric illnesses, and brain tumors, remain among the most challenging areas in drug discovery. Despite substantial advances in target identification and medicinal chemistry, the clinical success rate of CNS drug candidates remains strikingly low [1,2].

**Figure 1:**
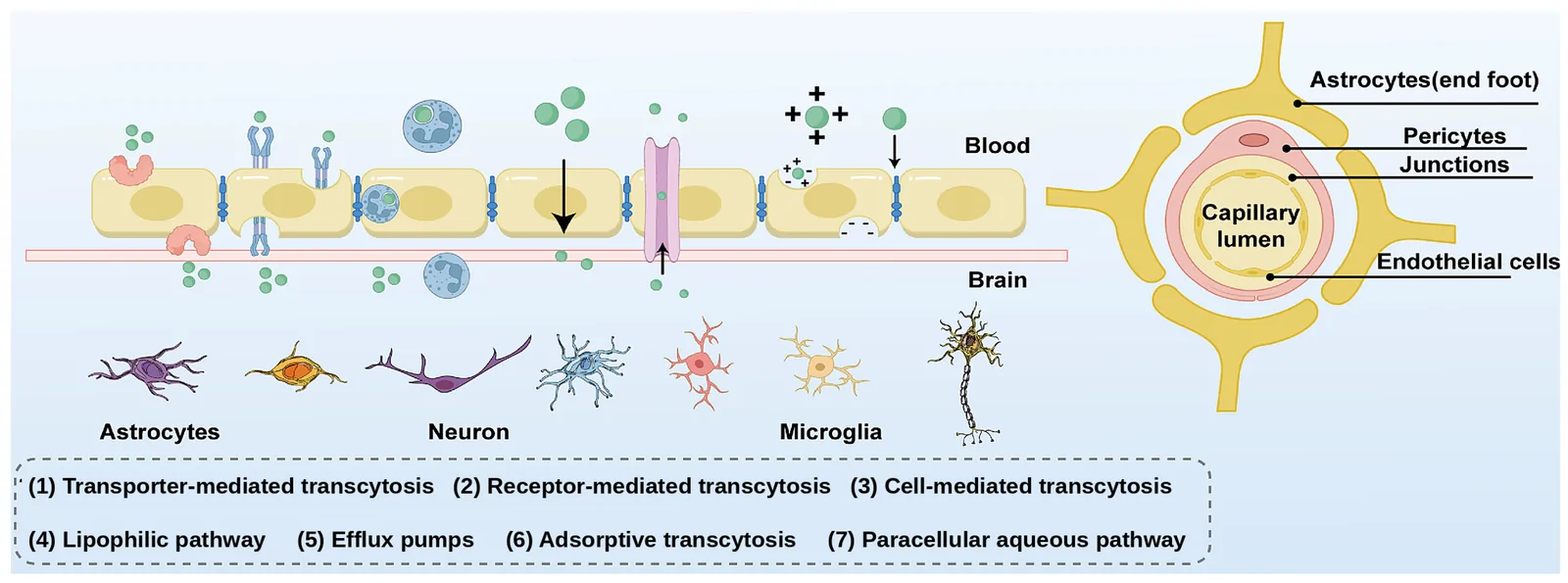
Overview of molecular transport routes across the blood–brain barrier (BBB). The BBB is composed of endothelial cells connected by tight junctions and supported by pericytes and astrocytes, forming a selective barrier between the systemic circulation and the brain parenchyma. Molecules traverse the BBB through multiple pathways: (1) transporter-mediated transcytosis, (2) receptor-mediated transcytosis, (3) cell-mediated transcytosis, (4) passive transcellular diffusion, (5) active efflux via transporters, (6) adsorptive transcytosis, and (7) paracellular aqueous diffusion.

A central and persistent bottleneck is the blood–brain barrier (BBB), which is formed by brain microvascular endothelial cells connected by tight junctions and supported by pericytes and astrocytes. Together, these components create a highly selective barrier with extremely low paracellular permeability [3] that separates the systemic circulation from the brain parenchyma [4]. Molecular transport across the BBB occurs through multiple parallel routes, including passive diffusion, several forms of transcytosis, and active efflux mechanisms (Figure 1). While some small molecules cross the BBB via passive transcellular diffusion, many are excluded because of unfavorable physicochemical properties or are actively removed by efflux transporters such as P-glycoprotein (P-gp) and breast cancer resistance protein (BCRP) [5,6]. It has been estimated that a large majority of small-molecule drug candidates fail to achieve sufficient brain exposure because of inadequate BBB penetration [7], contributing to high attrition during late-stage development.

The combination of structural restriction and active efflux makes BBB permeability a multifactorial and inherently difficult-to-predict property [8]. The coexistence of multiple transport routes, each governed by distinct physicochemical and biological determinants, highlights the intrinsic complexity of BBB permeability and contributes to the limited interpretability of many current predictive models. Over the past decades, numerous chemical and experimental strategies have been developed to overcome the BBB constraint [9]. From a medicinal chemistry perspective, optimization of physicochemical properties—including reduction in polar surface area, limitation of hydrogen-bonding capacity, and tuning of lipophilicity—remains the most widely applied approach to enhance passive BBB diffusion [10,11]. Such heuristic rules, however, often conflict with requirements for potency, selectivity, or safety, and their applicability varies across chemical classes [12].

In vitro models are commonly used to evaluate different aspects of BBB penetration. PAMPA-BBB primarily assesses apparent passive membrane permeability, whereas cell-based models such as MDR1-MDCK can provide information on transporter-mediated efflux, and in situ brain perfusion offers a more integrated assessment of brain uptake [13,14]. Although these assays provide valuable mechanistic insight, they are labor-intensive, costly, and impractical for large-scale screening of modern ultra-large compound libraries [15]. Alternative delivery strategies, including prodrug design, nanoparticle-mediated transport, and receptor-mediated transcytosis, have also been explored [16,17,18]; however, these approaches are typically target- or modality-specific and difficult to generalize across diverse small-molecule spaces. Consequently, there is a strong need for robust computational tools capable of prioritizing BBB permeability efficiently and at scale [19].

Various in silico models have been proposed for BBB-permeability prediction, spanning classical quantitative structure–activity relationship (QSAR) approaches to contemporary machine learning and deep learning methods [20,21]. Traditional models based on physicochemical descriptors and algorithms such as support vector machines, random forests, and gradient boosting have reported strong performance on curated benchmark datasets [22]. More recently, neural network-based architectures, including recurrent neural networks and graph neural networks, have further improved predictive accuracy by learning structure–property relationships directly from molecular representations [23].

Most of these models are trained and evaluated on relatively small and chemically homogeneous datasets that often overrepresent BBB-permeable compounds and may not reflect the distribution encountered in real-world screening libraries [24]. In practice, BBB-permeable molecules constitute only a small fraction of drug-like chemical space, yet many publicly available datasets adopt artificially balanced or even permeability-biased class distributions [25]. This mismatch can inflate apparent performance metrics and reduce generalization when models are applied prospectively [24]. Meanwhile, negative (BBB-impermeable) samples are frequently underrepresented, weakly annotated, or confined to narrow chemical domains, limiting a model’s capacity to reliably reject non-permeable compounds—a function that is essential for large-scale library filtering [26]. Together, these limitations constrain the practical utility of many existing BBB-prediction tools in CNS drug discovery pipelines.

In this work, we address these challenges by developing DeepBBB, a graph-based screening workflow designed for practical BBB-permeability prioritization and large-scale compound filtering. Rather than emphasizing architectural novelty, we treated data representativeness—particularly the composition of the negative class—as a primary design variable [27]. To construct a more strongly negative-enriched training configuration, we expanded the negative class using non-CNS agents from DrugBank [28] and commercially available background libraries, treating these as presumed-negative or unlabeled background compounds rather than experimentally confirmed BBB-negative molecules. Based on this curated dataset, we built graph neural network models for both binary classification and quantitative regression. By combining high-specificity classification with regression-based prioritization, DeepBBB enables the efficient filtering of ultra-large chemical libraries and the construction of BBB-focused screening datasets. We further evaluate the workflow through prospective in vitro PAMPA-BBB testing and an illustrative target-based CNS prioritization case study.

Graph-based models have previously been applied to BBB-permeability prediction, and no architectural priority is claimed here. Kato and colleagues built PAMPA-BBB models from approximately 2000 internally measured compounds using a message-passing graph convolutional network [29]; Jing and colleagues benchmarked graph convolutional variants for binary BBB classification on 1924 molecules [23]; and Lahoti and Bhende reported a gated message-passing network with a transformer readout trained on the literature-derived MoleculeNet BBBP labels [30]. The distinction of the present study lies in treating negative/background-class composition as an operational screening variable, applying the resulting filters to an approximately 1.7-billion-compound make-on-demand space, constructing three explicitly defined BBB-focused libraries, and carrying the workflow through prospective PAMPA-BBB evaluation.

## 2. Results

### 2.1. DeepBBB Workflow and Data-Composition Design

**Figure 2:**
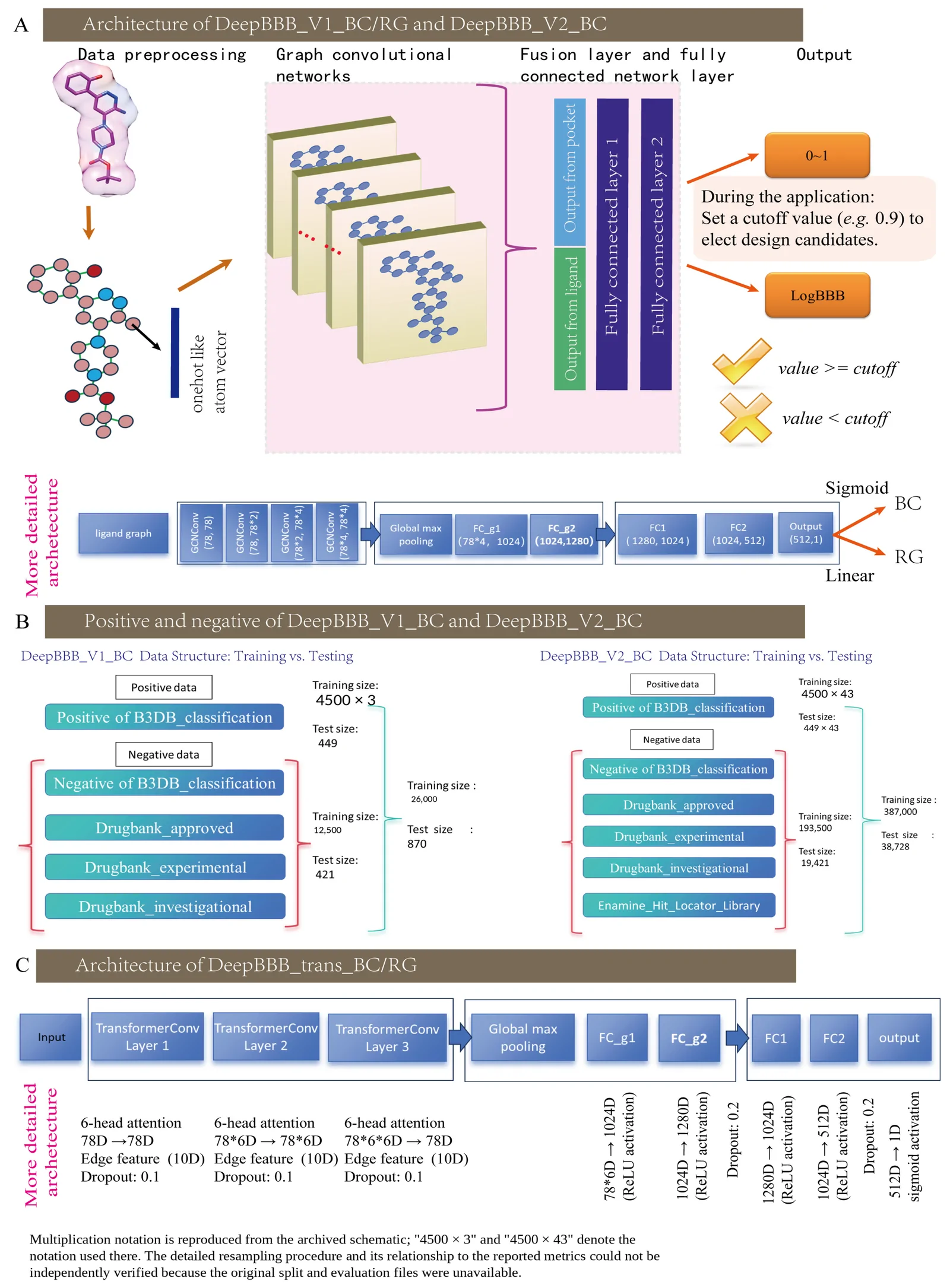
Schematic overview of DeepBBB models: architectures and training-data composition. (**A**) Architecture of DeepBBB_V1_BC/RG and DeepBBB_V2_BC, which share an identical network structure and differ in training-data composition (see panel (**B**)). (**B**) Training and testing data composition. Positive samples derive from B3DB-classified BBB-permeable compounds. For DeepBBB_V1_BC, the negative class consists of B3DB BBB-impermeable compounds combined with non-CNS DrugBank entries (approved, experimental, investigational), treated as presumed negatives. For DeepBBB_V2_BC, the negative class is further expanded with unlabeled background compounds from the Enamine Hit Locator Library, increasing the size and diversity of the negative set. (**C**) Architecture of DeepBBB_trans_BC/RG, in which the GCN layers are replaced with graph-transformer modules; DeepBBB_trans_BC is trained on the same dataset as DeepBBB_V2_BC. (Original schematic retained; negative-class labels are interpreted as presumed-negative/unlabeled background compounds.) Multiplication notation is reproduced from the archived schematic. The detailed resampling procedure and its relationship to the reported metrics could not be independently verified because the original split and evaluation files were unavailable.

**Figure 3:**
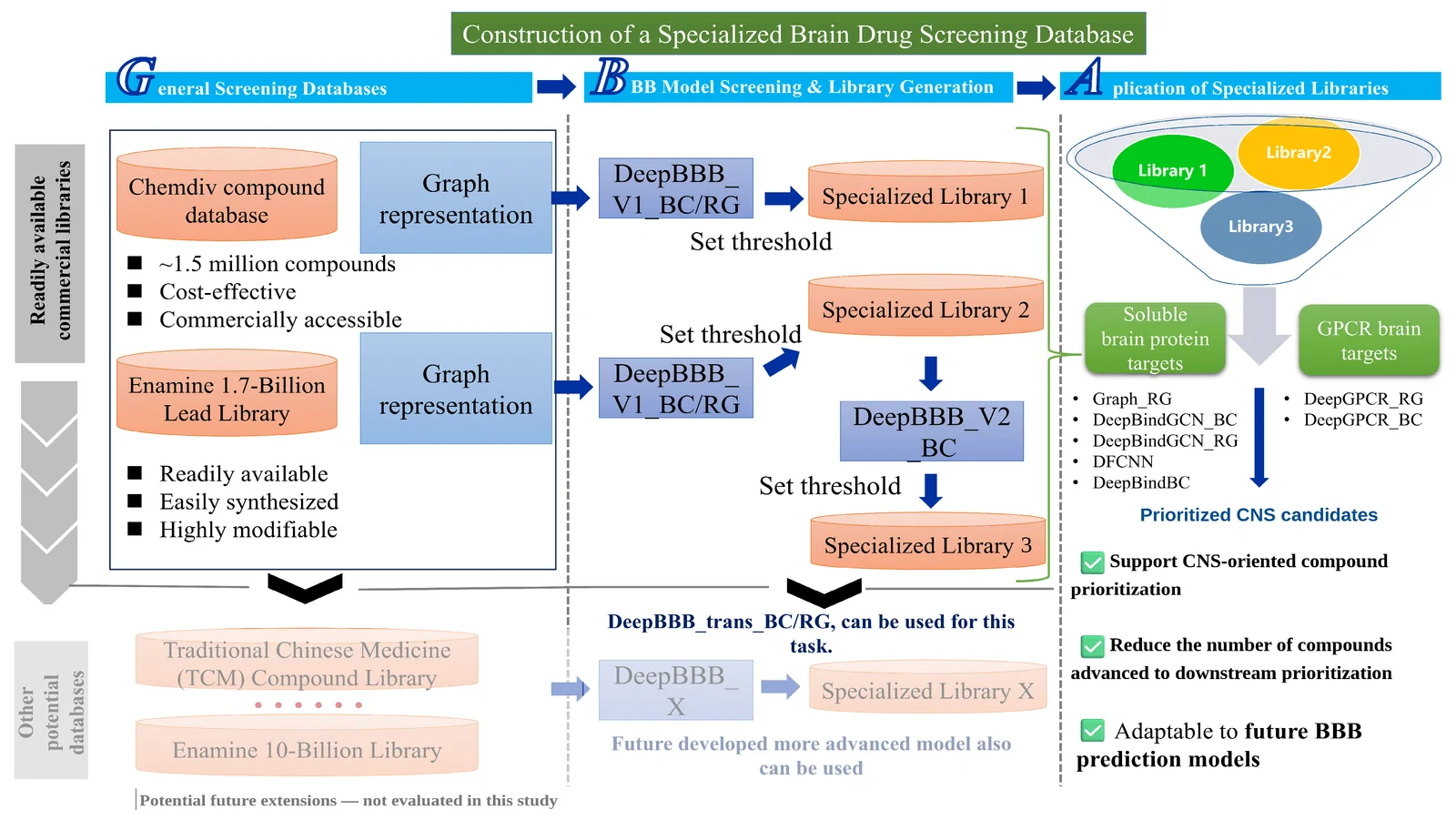
Strategy for constructing BBB-focused specialized libraries. Screening ChemDiv with DeepBBB_V1_BC/RG produced Specialized Library 1; applying the same filters to the Enamine REAL lead-like collection produced Specialized Library 2; and adding DeepBBB_V2_BC as a joint filter produced Specialized Library 3. The lower row indicates potential future extensions to additional source collections and future model variants; these were not screened in the present study, and no results are reported for them.

DeepBBB is organized as a modular workflow (Figure 1, Figure 2 and Figure 3): graph-based BBB-permeability models are trained on B3DB with a deliberately negative-enriched class composition (Figure 2), applied to commercial libraries to construct BBB-focused screening sets (Figure 3), profiled for chemical-space characteristics, and then evaluated prospectively by PAMPA-BBB. The principal design variable is the composition of the negative class: DeepBBB_V1 uses B3DB negatives augmented with non-CNS DrugBank compounds (operational ratio ~1:6), whereas DeepBBB_V2 adds Enamine background compounds (operational ratio ~1:43) to act as a more conservative filter during large-scale screening.

### 2.2. Archived Original Test-Split Summary Metrics

**Figure 4:**
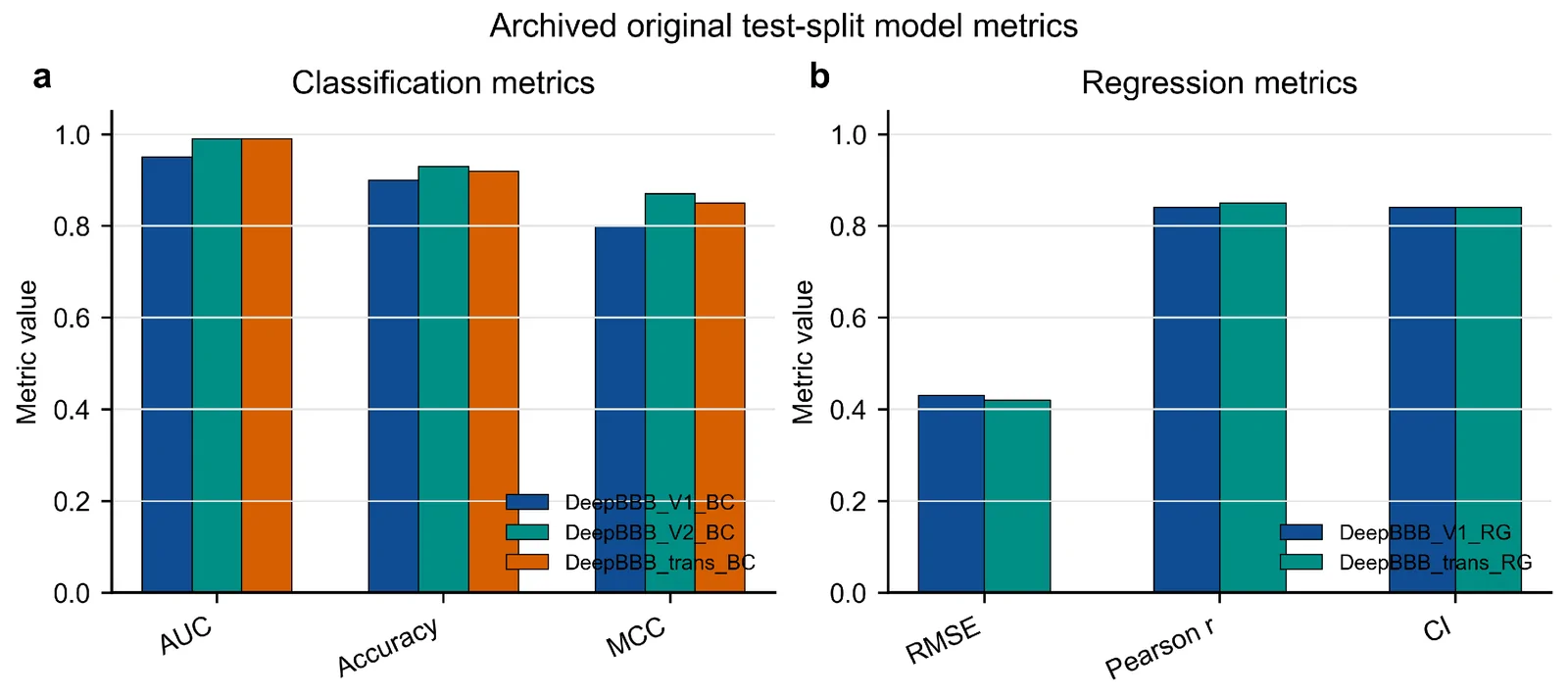
Archived original test-split model-metric summary (redrawn). (**a**) Archived classification summary metrics for DeepBBB_V1_BC, DeepBBB_V2_BC, and DeepBBB_trans_BC evaluated by AUC, accuracy (ACC), and Matthews correlation coefficient (MCC): AUC 0.95/0.99/0.99, ACC 0.90/0.93/0.92, MCC 0.80/0.87/0.85. (**b**) Archived regression summary metrics for DeepBBB_V1_RG and DeepBBB_trans_RG evaluated by RMSE (0.43/0.42), Pearson r (0.84/0.85), and concordance index (0.84/0.84). Values are archived retrospective metrics from the original test split; the figure does not reconstruct ROC, precision–recall, calibration, or scaffold-split performance.

Archived original test-split summary metrics are reported in Figure 4 and Tables S1–S5 solely to document the historical models used in the screening workflow (Figure 4a; Tables S1–S3). DeepBBB_V1_BC, DeepBBB_V2_BC, and DeepBBB_trans_BC achieved AUC values of 0.95, 0.99, and 0.99, respectively, with accuracies of 0.90, 0.93, and 0.92 and Matthews correlation coefficients (MCC) of 0.80, 0.87, and 0.85. For regression (Figure 4b; Tables S4 and S5), DeepBBB_V1_RG achieved an RMSE of 0.43, a Pearson correlation coefficient of 0.84, and a concordance index (CI) of 0.84, while DeepBBB_trans_RG achieved an RMSE of 0.42, a Pearson r of 0.85, and a CI of 0.84. The GCN-based regressor showed somewhat more stable training behavior than the transformer-based variant and was used as the preferred quantitative model.

These values are reported as archived retrospective summary metrics on the original test split. Because the positive class is relatively small and the training and test sets were drawn from the same underlying source, the test set may represent a comparatively favorable evaluation scenario; the metrics are therefore not presented as scaffold-split, external-validation, or prospective-generalization performance, and the training-epoch stability is treated as a descriptive observation rather than proof of the absence of overfitting. For this reason, the prospective PAMPA-BBB campaigns are treated as operational evaluations of the end-to-end workflow rather than as validation of model generalization or superiority over baseline selection strategies. The archived schematic contains multiplication notation for the DeepBBB_V2_BC test partition, but its relationship to metric calculation could not be independently verified; this is an additional reason to treat the corresponding values descriptively.

### 2.3. Construction of BBB-Focused Specialized Libraries

Applying the DeepBBB filters to commercial chemical space produced three progressively refined libraries (Table 1): Specialized Library 1 (21,991 ChemDiv compounds; V1_BC ≥ 0.99, RG ≥ 0.3), Specialized Library 2 (4,808,885 Enamine REAL lead-like compounds; V1_BC = 1.0, RG ≥ 0.5), and Specialized Library 3 (151,790 Enamine REAL lead-like compounds; V1_BC = 1.0, RG ≥ 0.5, V2_BC = 1.0). The successive addition of DeepBBB_V2_BC as a joint constraint substantially reduced the retained set, consistent with its intended role as a more selective operational filter.

### 2.4. Physicochemical Profiles of the Selected Libraries

**Figure 5:**
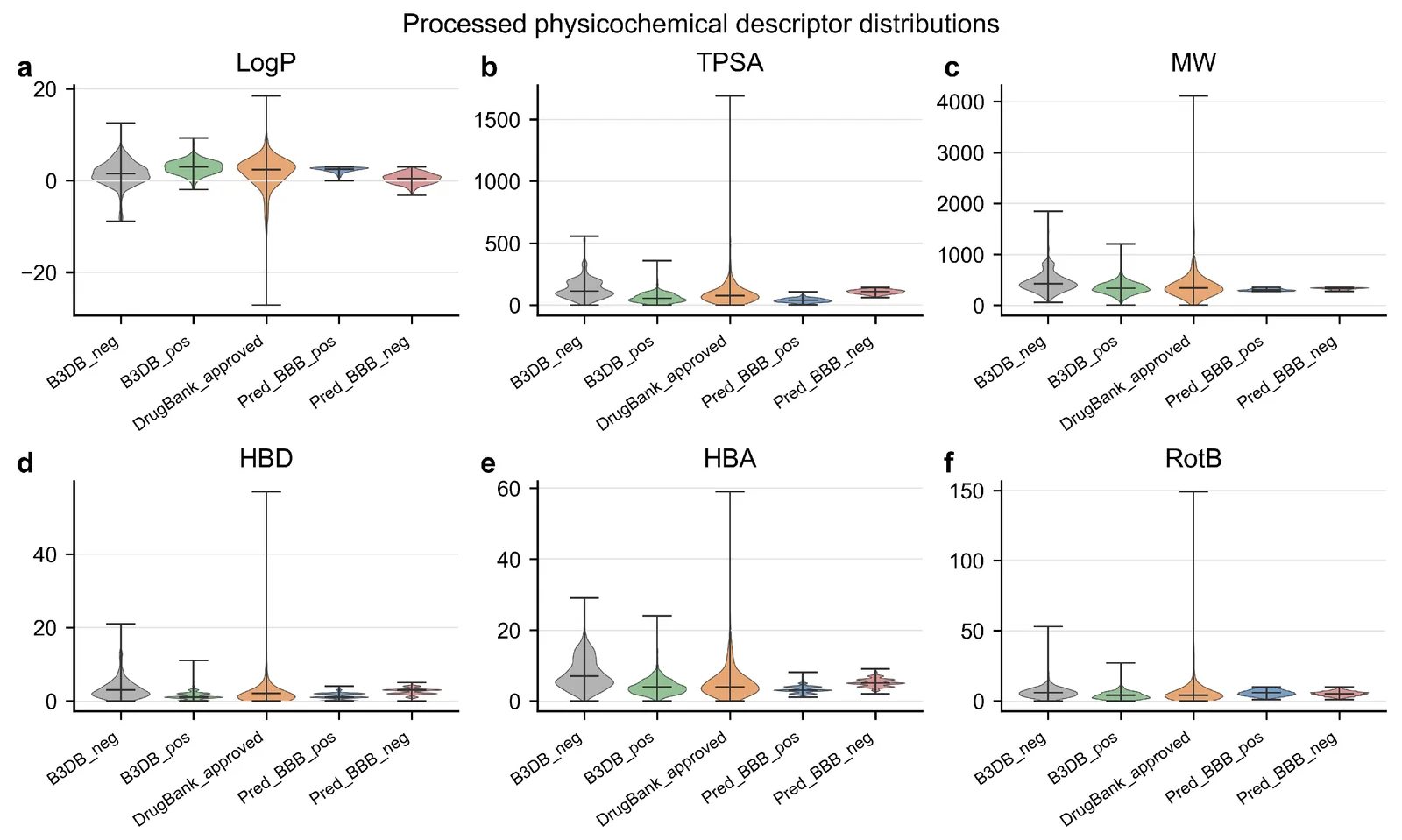
Physicochemical descriptor profiles of the DeepBBB-derived BBB-focused library. (**a**–**f**) Distributions of logP, TPSA, molecular weight (MW), hydrogen-bond donors (HBD), hydrogen-bond acceptors (HBA), and rotatable bonds (RotB) for B3DB_neg, B3DB_pos, DrugBank_approved, Pred_BBB_pos, and Pred_BBB_neg, redrawn from available processed descriptor tables. Pred_BBB_pos occupies a comparatively compact BBB-compatible region overlapping B3DB_pos and separated from B3DB_neg and DrugBank_approved. The archived pairwise and nearest-neighbor Tanimoto-similarity panels from the original figure are retained as Supplementary Figure S1 because the per-pair similarity matrices were not available locally for full redrawing.

Across the six descriptors, the predicted BBB-permeable set (Pred_BBB_pos; Specialized Library 3) occupied a comparatively compact, BBB-compatible region (Figure 5a–f). Relative to the more polar and hydrogen-bond-rich B3DB_neg set and the broader, indication-agnostic DrugBank-approved collection, Pred_BBB_pos exhibited moderate-to-high lipophilicity and lower polarity, closely resembling B3DB_pos. In the available descriptor summaries, the predicted-positive set showed a low mean TPSA (~39 Å^2^) and low mean HBD count (~1.1), whereas the predicted non-permeable set (Pred_BBB_neg) showed higher TPSA (~107 Å^2^) and greater hydrogen-bonding capacity and flexibility—chemotype characteristics generally disfavored for passive brain penetration (Table S6).

Library diversity was assessed using Tanimoto similarity. Pairwise similarity within Pred_BBB_pos was comparable to that of B3DB_pos and greater than that of Pred_BBB_neg, while DrugBank-approved showed the widest similarity distribution, consistent with its heterogeneous clinical composition (Supplementary Figure S1). Nearest-neighbor analysis indicated that most Pred_BBB_pos molecules shared only low-to-moderate similarity with their closest counterparts in B3DB_pos or DrugBank-approved and even lower similarity with B3DB_neg, while within-set nearest-neighbor similarity shifted higher (Supplementary Figure S1), reflecting recurrent permeability-favorable motifs within an otherwise diverse set. These archived similarity panels are retained in Supplementary Figure S1 because the per-pair similarity matrices were not available locally for full redrawing. These scaffold and similarity statistics describe the constructed library and do not establish scaffold novelty for the prospectively tested compounds.

### 2.5. Similarity, Fragment, and Scaffold Summaries

**Figure 6:**
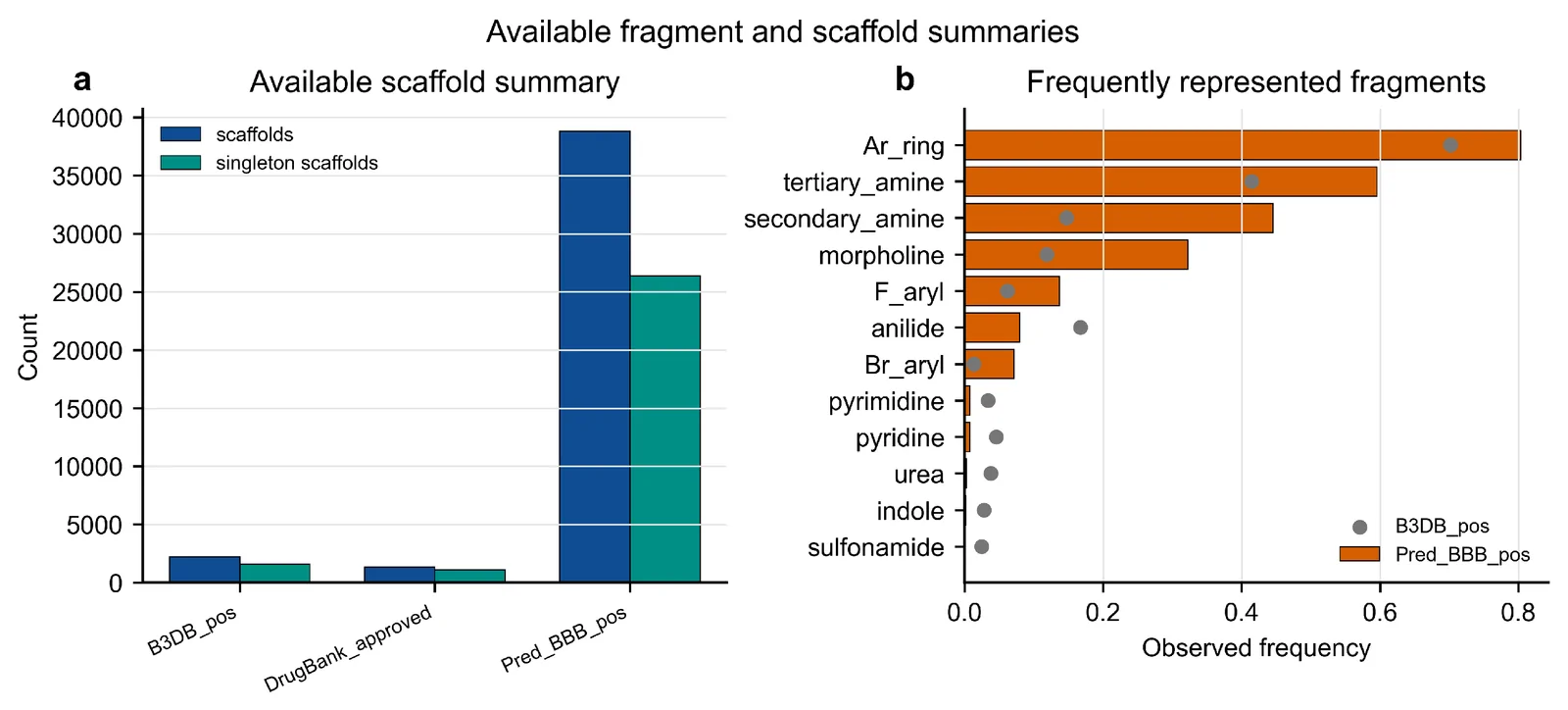
Available fragment and scaffold summaries for the DeepBBB-derived BBB-focused library. (**a**) Bemis–Murcko scaffold counts and singleton-scaffold counts for B3DB_pos, DrugBank_approved, and Pred_BBB_pos, redrawn from available scaffold summary tables. (**b**) Frequently represented fragments in Pred_BBB_pos compared with B3DB_pos, based on available fragment-summary tables. “Frequently represented” denotes observed fragment frequency rather than a newly reconstructed formal enrichment test. The original UMAP, fragment-heatmap, and scaffold-novelty panels are retained as Supplementary Figure S2.

An archived UMAP projection is retained in Supplementary Figure S2 because the underlying coordinates were not available locally; in that projection, Pred_BBB_pos clustered near B3DB_pos while extending into adjacent regions of chemical space less represented in current reference datasets. In the redrawn main-text summary, fragment-summary tables indicated that aryl rings, tertiary amines, secondary amines, morpholines, aryl fluorides, anilides, and aryl bromides were frequently represented in Pred_BBB_pos relative to B3DB_pos (Figure 6b; Table S7); these motifs were most frequent in Pred_BBB_pos, intermediate in DrugBank-approved and B3DB_pos, and least frequent in Pred_BBB_neg, consistent with the model favoring polarizable and moderately basic substructures commonly associated with brain penetration.

At the scaffold level, Pred_BBB_pos contained substantially more Bemis–Murcko frameworks than the smaller B3DB and DrugBank reference sets. In the available scaffold summary, Pred_BBB_pos comprised 38,807 distinct Bemis–Murcko scaffolds across 151,729 molecules, including 26,412 singleton scaffolds, of which approximately 99.6% were not present in B3DB_pos (Figure 6a; Table S7). This library-level scaffold summary indicates that the predicted-positive library is not limited to scaffolds represented in B3DB_pos. It should not be interpreted as scaffold-level validation of the prospectively tested PAMPA-BBB-positive compounds, for which machine-readable structures matching the Table 2 identifiers were not available in the local archive.

### 2.6. Prospective PAMPA-BBB Evaluation: First Campaign

**Figure 7:**
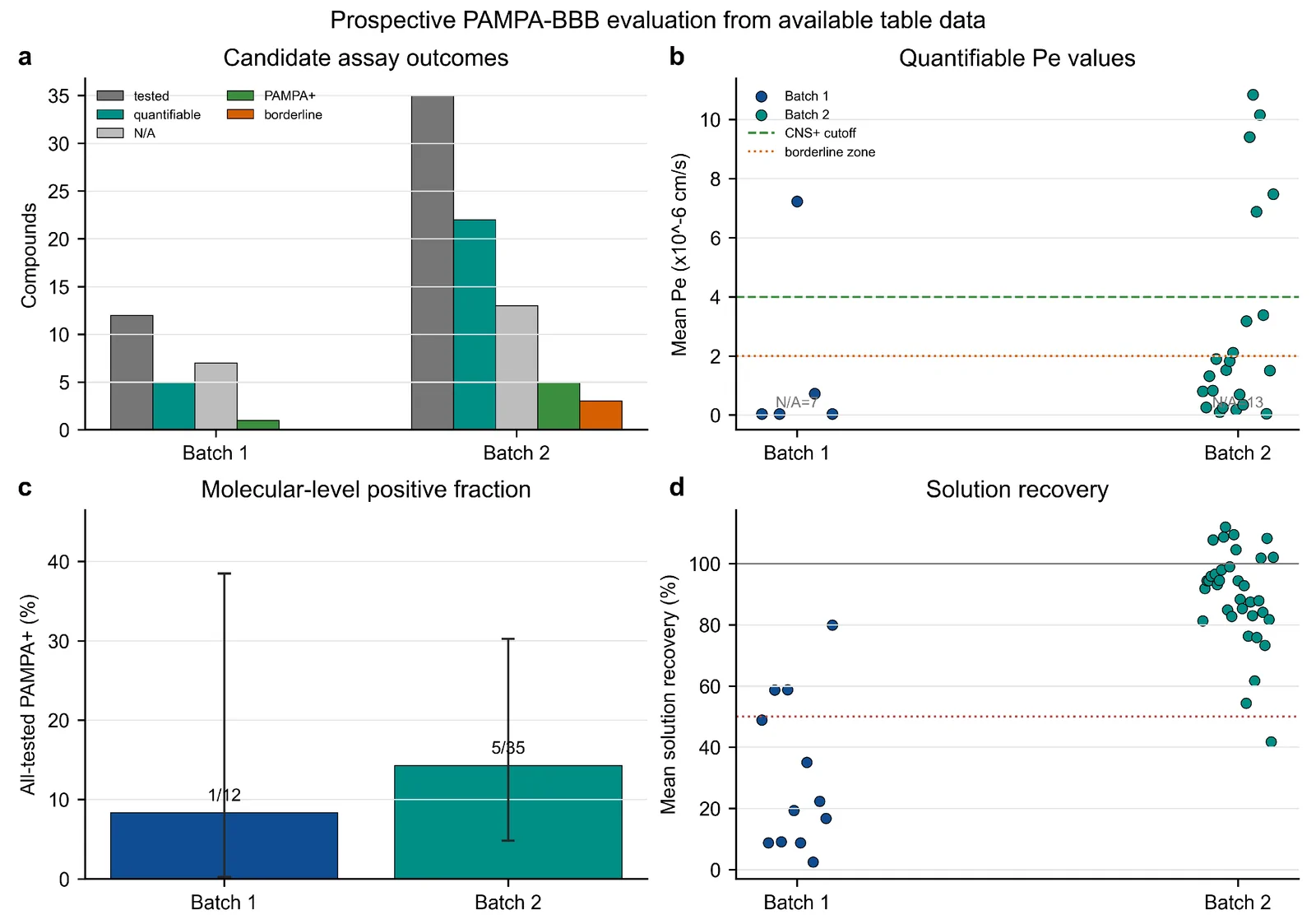
Prospective PAMPA-BBB evaluation (redrawn from available table data). (**a**) Candidate assay outcomes for the first (12 candidates) and second (35 candidates) campaigns, separating all-tested denominators, (**b**) quantifiable Pe values, (**d**) solution recovery, not-quantifiable (N/A) entries, and exact binomial 95% confidence intervals for the all-tested (**c**) molecular-level PAMPA-BBB-positive fractions (1/12 = 8.3%; 5/35 = 14.3%). N/A values are not plotted as Pe = 0.

For the first campaign, WuXi AppTec evaluated the top model-predicted compounds using its standardized PAMPA-BBB protocol. Twelve of these candidate compounds were commercially available, procured, and tested (Table 3). One compound (FF01-5568) was PAMPA-BBB-positive, with a Pe of 7.23 × 10^−6^ cm·s^−1^, corresponding to an all-tested molecular-level positive fraction of 1/12 = 8.3% (exact 95% CI 0.2–38.5%); seven compounds were not quantifiable. This result demonstrates that the end-to-end workflow can identify a PAMPA-BBB-positive compound from a model-prioritized candidate set; however, because no matched comparator was tested, it does not establish that DeepBBB outperforms random selection or alternative prioritization strategies (Figure 7).

### 2.7. Prospective PAMPA-BBB Evaluation: Second Campaign with Corrected Denominator

In the second campaign, 35 commercially available top-ranked candidate compounds prioritized using DeepBBB_V1_BC/RG together with DeepBBB_V2_BC were evaluated using the in-house PAMPA-BBB protocol (Table 2). Five compounds were PAMPA-BBB-positive (Pe > 4 × 10^−6^ cm·s^−1^: Z9837060450, Z9837060457, Z9837060459, Z9837060461, and Z8242448685; Pe 6.88–10.84 × 10^−6^ cm·s^−1^), three were borderline, and 13 were not quantifiable. The all-tested molecular-level PAMPA-BBB-positive fraction was 5/35 = 14.3% (exact 95% CI 4.8–30.3%) (Figure 7). The N/A outcomes were reported separately. Although nominal solution recovery varied across compounds, the available summary records do not establish a single cause for the N/A classifications.

The second-campaign fraction (14.3%) was numerically higher than the first (8.3%). However, the two campaigns differed in compound source (ChemDiv vs. Enamine REAL lead-like), selection strategy (V1 only vs. V1 + V2), assay setting (external vs. in-house), procurement constraints, and likely chemotype composition. The difference therefore cannot be causally attributed to DeepBBB_V2 and is reported descriptively. The all-tested denominator is the 35 candidate compounds listed in Table 2, giving 5/35 = 14.3%, with not-quantifiable compounds reported separately as summarized in Supplementary Methods S2.

### 2.8. Structure/Scaffold Interpretation of Prospective Positives

Five PAMPA-BBB-positive compounds were identified at the molecular level in the second campaign. Machine-readable structures matching the Table 2 compound identifiers were not available in the local archive; consequently, Bemis–Murcko scaffold assignment, pairwise Tanimoto clustering, scaffold counting, and nearest-neighbor similarity to training-set BBB-positive compounds were not computed for these five compounds. We therefore report five PAMPA-BBB-positive molecules at the molecular level and do not claim that they represent five distinct or structurally diverse scaffolds; chemotype-level interpretation is deferred to author-supplied structures. Model scores for the 33 prioritized Enamine selection records are given in Table S8; these archived selection records are not used as the assay denominator, which is defined by the 35 tested candidate compounds listed in Table 2. Canonical structures and model scores for these 33 archived selection records are provided as Supplementary Table S9; the records are keyed by Enamine synthon-style identifiers, and no mapping between those identifiers and the catalog numbers of the 35 assayed compounds was available, so model scores could not be linked to individual assay outcomes.

### 2.9. Computational Downstream Prioritization Using Human Glutaminyl Cyclase as an Illustrative CNS Target

**Figure 8:**
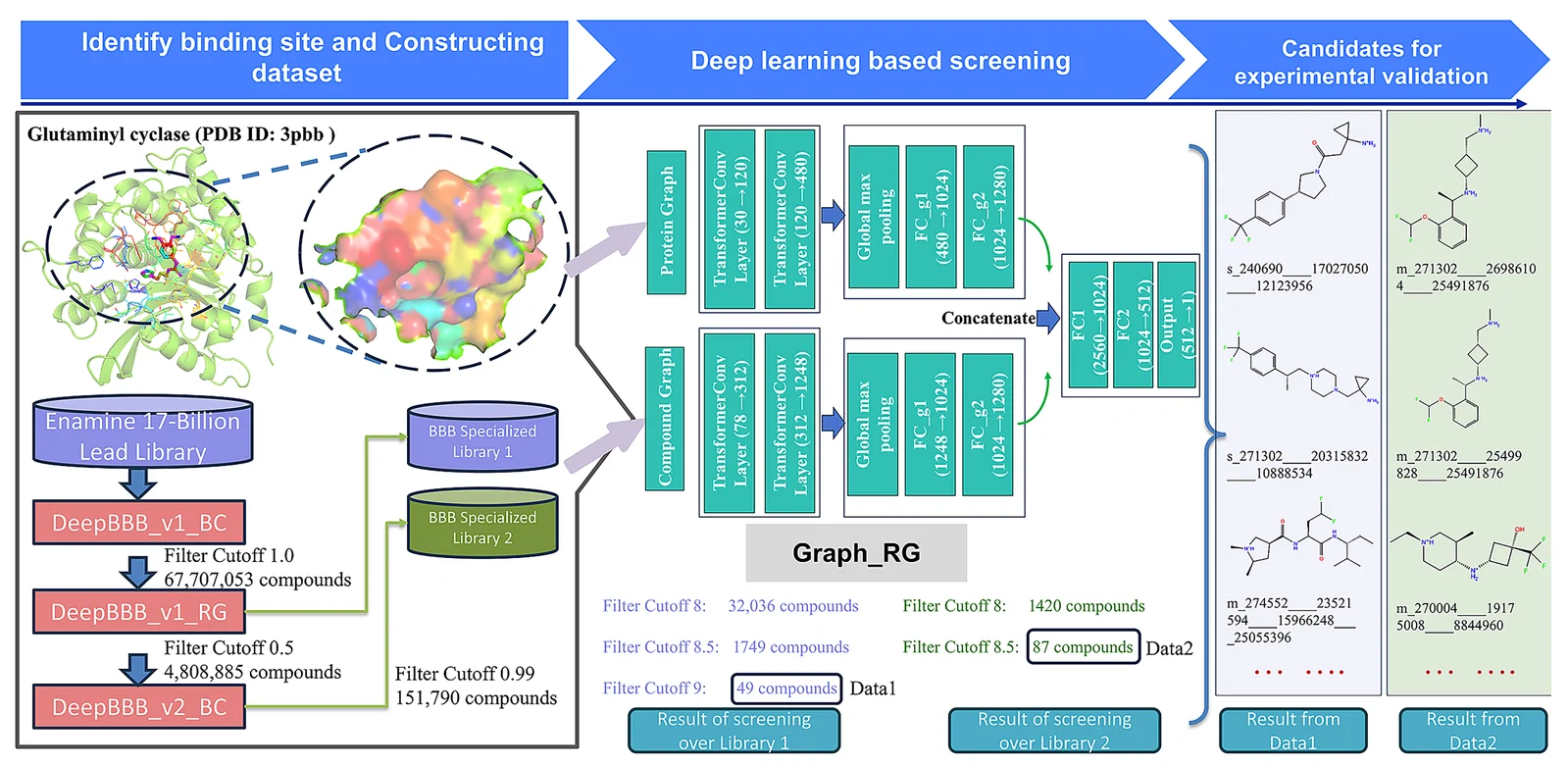
DeepBBB–Graph_RG pipeline for CNS-focused computational prioritization. The Enamine REAL lead-like library is filtered by DeepBBB_V1_BC and DeepBBB_V1_RG to generate Specialized Library 2 and, with joint DeepBBB_V2_BC, the more selective Specialized Library 3. Both libraries are ranked against human glutaminyl cyclase (PDB 3PBB) using the Graph_RG affinity model, narrowing millions of BBB-compatible molecules to compact panels of top-ranked putative binders. Representative high-scoring structures are shown on the right. This is a computational prioritization illustration and does not include experimental target-binding results.

To illustrate the application of BBB-focused libraries to target-based prioritization, human glutaminyl cyclase (PDB ID 3PBB) was used as a representative CNS target (Figure 8). Starting from the Enamine REAL lead-like collection, DeepBBB_V1_BC was applied with a stringent permeability threshold (score = 1.0), yielding approximately 67.7 million predicted BBB-permeable candidates; these were further filtered using DeepBBB_V1_RG (predicted logBBB ≥ 0.5) to define Specialized Library 2 (4,808,885 compounds). In parallel, joint application of DeepBBB_V2_BC produced the more selective Specialized Library 3 (151,790 stringently filtered candidates). Both libraries were ranked against glutaminyl cyclase using the Graph_RG affinity model. Sequential filtering by Graph_RG score progressively reduced the candidate space from millions of BBB-compatible molecules to compact panels of top-ranked putative binders suitable for purchase and experimental evaluation. This case study illustrates a modular workflow in which BBB compatibility is applied as an upstream constraint before target-based ranking; it is computational and does not include experimental target-binding results.

## 3. Discussion

We present DeepBBB, a graph-based, data-composition-aware screening workflow developed to prioritize BBB permeability and to construct BBB-focused virtual libraries enriched in predicted BBB-compatible candidates. By combining curated B3DB data with a systematically expanded, presumed-negative class derived from non-CNS DrugBank compounds and Enamine background molecules, the workflow is designed to act as a conservative filter for large-scale screening. The archived model summaries document the historical models used in the scalable filtering pipeline but are not treated as evidence of predictive generalization.

One notable observation is the influence of training-data composition on model behavior. Although all three classification encoders performed similarly on the archived B3DB test split, the GCN-based regressor showed greater training stability than the transformer-based variant. This suggests that, under current data constraints, the representativeness and balance of annotated datasets may matter as much as encoder choice. When the negative class was expanded to create a more strongly negative-enriched configuration, DeepBBB_V2_BC functioned as a more selective filter during large-scale screening. DeepBBB_V2_BC reduced the retained virtual library as intended. Because the campaigns differed in compound source, selection strategy, procurement constraints, and assay setting, their outcomes do not establish whether the additional filter improved prospective enrichment.

Physicochemical and chemotype profiling support the plausibility of the selected libraries. The predicted BBB-positive space occupies a defined region of moderate molecular weight, balanced lipophilicity, and constrained hydrogen-bonding capacity, closely aligning with B3DB-positive compounds. The available library-level scaffold summary suggests that the predicted-positive library is not limited to scaffolds represented in B3DB_pos, while recurrent permeability-associated features (e.g., aryl halides and morpholines) remain frequently represented. This is a descriptive library-level summary of processed data and is not a scaffold-level validation of the model or of the prospectively tested compounds.

The utility of these BBB-focused libraries was further examined by integration with a target-specific affinity model. Using human glutaminyl cyclase as an illustrative target, the DeepBBB filters reduced the Enamine REAL lead-like collection to manageable sub-libraries that could be prioritized using Graph_RG. This cascade separates BBB compatibility from target binding as sequential constraints, offering a modular structure for CNS-oriented discovery pipelines. As implemented here, the case study is computational; experimental confirmation of target binding for prioritized candidates is a natural next step.

Prospective in vitro testing provided an operational assessment of the end-to-end workflow. The identification of PAMPA-BBB-positive compounds in both external and internal campaigns—including compounds such as FF01-5568—shows that the workflow can surface compounds with measurable apparent passive permeability; however, the absence of a matched comparator prevents attribution of these outcomes specifically to DeepBBB or claims of superiority over baseline selection strategies. PAMPA-BBB measures apparent passive membrane permeability under the assay conditions; it does not measure P-gp or BCRP liability, active influx, receptor-mediated transport, unbound brain partitioning (Kp,uu), total brain-to-plasma partitioning (Kp,brain), or in vivo CNS exposure. Accordingly, PAMPA-BBB-positive compounds are described as having high apparent passive permeability under the assay conditions, and not as a measure of in vivo brain penetration or of transporter-mediated efflux. Prospectively generated measurements may in future be incorporated into iterative model-refinement and compound-selection cycles. More broadly, emerging adaptive artificial-intelligence frameworks that continuously incorporate new experimental observations into subsequent model updates offer a useful perspective on how screening workflows of this kind might be made more adaptable over time [31].

### Limitations

Several limitations should be acknowledged. First, the assumption that non-CNS drugs and commercial background compounds are uniformly BBB-impermeable, while operationally useful for negative-class expansion, introduces label noise; refinement of negative annotations with higher-resolution permeability data would improve calibration. Second, the reported model metrics correspond to an archived original test split: per-sample predictions, exact split assignments, training checkpoints, and seeds were not re-derived here, so scaffold-split validation and train–test leakage auditing were not performed and remain open. Third, raw plate-level PAMPA replicate data were not re-analyzed, so replicate-level dispersion statistics are not reported, and small-panel recovery variability complicates precise hit-rate estimation. The high proportion of not-quantifiable outcomes also reduces practical screening efficiency by lowering the number of interpretable results obtained per purchased and tested compound. Fourth, machine-readable structures for the second-campaign-tested compounds, including the five positives, were not available locally, precluding scaffold-level and Tanimoto analysis of the prospective positives. Fifth, the Graph_RG/glutaminyl cyclase analysis is a computational prioritization case study without experimental target-binding measurements. Finally, the models use composite permeability endpoints and do not explicitly distinguish passive diffusion from transporter-mediated processes, which contribute differently to in vivo CNS exposure.

## 4. Materials and Methods

### 4.1. Evidence Boundary and Data Availability

This manuscript reports a workflow assembled from models, processed datasets, summary tables, and prospective assay results. To keep claims aligned with the supporting evidence, model-performance values are reported as archived retrospective metrics from the original training/test split; per-sample predictions, exact split assignments, training checkpoints, and random seeds were not re-derived here, so the reported metrics are not described as scaffold-split or external-validation performance. PAMPA-BBB outcomes are reported at the level of per-compound mean permeability and categorical assignment; raw plate-level replicate measurements were not re-analyzed, so no new standard deviations, replicate confidence intervals, or reproducibility statistics are introduced. Physicochemical, fragment, and scaffold analyses are based on available processed descriptor and summary tables. The original ultra-large screening databases (Enamine REAL, ChemDiv) and licensed third-party datasets (DrugBank) are not redistributed; reported library sizes are retained as summary counts. A complete account of data provenance and access routes is given in the Data Availability Statement and Supplementary Information.

### 4.2. Molecular Representation and Model Architecture

DeepBBB encompasses three variants: DeepBBB_V1_BC/RG, DeepBBB_V2_BC, and DeepBBB_trans_BC/RG. All variants share a common molecular representation in which compounds are encoded as atom–bond graphs, with atoms as nodes and bonds as edges, and each atom described by a 78-dimensional feature vector.

In DeepBBB_V1 and DeepBBB_V2, node features are processed through four stacked graph convolutional network (GCN) layers with ReLU activation. The first convolutional layer preserves the input dimensionality at 78, while subsequent layers increase the hidden dimensions to 156 and then 312, with the final convolutional layer maintaining a 312-dimensional representation. A global max-pooling operation across all nodes yields a fixed 312-dimensional molecular embedding. This embedding is passed through a shared fully connected backbone with layer dimensions of 312 → 1024 → 1280 → 1024 → 512. Two task-specific heads are defined on top of the shared backbone: a sigmoid-activated unit for binary classification (BBB+/BBB−), producing the sigmoid output score for BBB permeability, and a linear unit for regression of logBBB values. DeepBBB_V1_BC and DeepBBB_V2_BC share an identical network topology and differ only in the composition and class balance of their training data (Figure 2A).

To capture more complex intramolecular dependencies, DeepBBB_trans replaces the GCN stack with three graph-transformer blocks that apply multi-head self-attention over atom representations and incorporate learned edge embeddings (Figure 2C). The downstream global pooling layer, fully connected backbone, and classification/regression heads are kept identical to those of the GCN-based models, permitting a direct comparison between convolutional and transformer encoders under consistent training objectives. DeepBBB_trans_BC is trained on the same dataset as DeepBBB_V2_BC.

### 4.3. Data Curation and Presumed-Negative Augmentation

Training and test sets for both classification and regression were derived from the Blood–Brain Barrier Database (B3DB) [32]. For binary classification, compounds labeled BBB-permeable in B3DB_classification.tsv were assigned to the positive class, and compounds labeled BBB-impermeable were used as initial negatives. For regression, B3DB_regression.tsv was used, comprising compounds with experimentally measured logBBB values.

To broaden the chemical diversity of the negative class, DrugBank molecules annotated as approved, experimental, or investigational drugs without CNS indications or reported BBB penetration were incorporated and treated as presumed-negative (BBB-nonpermeable) decoys. This strategy is consistent with previously reported negative-class-expansion approaches in protein–ligand interaction prediction, including DeepBindBC [33] and DeepBindGCN [34]. For DeepBBB_V2_BC, the negative class was further expanded by incorporating compounds from the Enamine Hit Locator Library (Enamine_Hit_Locator_Library_200K_Set_plated) as unlabeled background compounds, substantially increasing the size and chemical diversity of the negative set (Figure 2B).

These augmentations changed the operational positive-to-negative ratio from approximately 1:6 in DeepBBB_V1 to approximately 1:43 in DeepBBB_V2. We refer to this as a negative-enriched, screening-like training configuration. We note explicitly that presumed-negative augmentation is an operational design choice intended to make the filter more conservative during large-scale screening; the resulting class ratio is not asserted to reproduce a measured physiological prevalence of BBB-permeable compounds, and DrugBank/Enamine background molecules are not experimentally confirmed BBB-negative.

### 4.4. Model Training and Archived Evaluation

All models were trained using mini-batches of 500 molecules and were implemented to run on both CPUs and GPUs. Binary cross-entropy loss was used for classification and mean squared error (MSE) loss for regression. Archived epoch-level performance summaries were available for the original training runs (Tables S1–S5; Figures S3 and S4; complete epoch-level trajectories are provided as Supplementary Data). In the original workflow, the application checkpoints retained for downstream screening were epoch 2000 for DeepBBB_V1_BC, epoch 100 for DeepBBB_V2_BC and DeepBBB_trans_BC, epoch 2000 for DeepBBB_V1_RG, and epoch 200 for DeepBBB_trans_RG. Because the local archive did not contain validation-set histories, split assignments, random seeds, or model checkpoints, these checkpoint-level metrics are reported descriptively as archived original test-split summaries and are not used here to claim independently optimized model selection. Because per-sample predictions, split assignments, seeds, and checkpoints were not re-derived in the present work, receiver-operating-characteristic reconstruction, calibration analysis, scaffold-split evaluation, and train–test leakage auditing were not performed here and are identified as priorities for future data-complete reanalysis. The archived data-composition schematic records multiplication notation for the DeepBBB_V2_BC-positive class in both the training and test partitions, and the displayed totals are arithmetically consistent with replicated rows. However, because the underlying split and evaluation files were not recoverable, the exact resampling procedure and its relationship with the reported metrics could not be independently verified. The DeepBBB_V2_BC and graph-transformer values are therefore treated only as unaudited archived summaries. Because per-compound predictions were not available, the sigmoid outputs of the classifiers should not be interpreted as calibrated probabilities, and any score cutoff used in this work functions as an operational ranking device. The graph convolutional configuration was retained for downstream deployment on operational grounds, including expected lower inference cost and simpler billion-scale deployment; no claim is made that it was more accurate than the graph-transformer variant.

### 4.5. Construction of BBB-Focused Screening Libraries

A BBB-focused screening database was constructed by applying the trained DeepBBB models to commercially available small-molecule libraries, including the ChemDiv collection (~1.5 million compounds) and the Enamine REAL lead-like collection (~1.7 billion compounds) (Figure 3). Each molecule was converted into a molecular graph and evaluated using DeepBBB_V1_BC, DeepBBB_V1_RG, and DeepBBB_V2_BC. Compounds with model scores for the BBB-permeable class above the defined operational score cutoffs were retained as putative BBB-positive candidates, and DeepBBB_V1_RG logBBB estimates were used to prioritize molecules predicted to combine favorable permeability with acceptable physicochemical profiles. Using this workflow, three specialized BBB-focused libraries were generated (numbering as used throughout this manuscript and summarized in Table 1):

- **Specialized Library 1**—21,991 compounds obtained by screening ChemDiv with DeepBBB_V1_BC/RG (thresholds V1_BC ≥ 0.99 and RG ≥ 0.3).
- **Specialized Library 2**—4,808,885 compounds derived from the Enamine REAL lead-like library using DeepBBB_V1_BC/RG with more stringent thresholds (V1_BC = 1.0 and RG ≥ 0.5).
- **Specialized Library 3**—151,790 compounds generated by jointly applying DeepBBB_V1_BC/RG and DeepBBB_V2_BC to the Enamine REAL lead-like library (thresholds V1_BC = 1.0, RG ≥ 0.5, and V2_BC = 1.0).

Together, these libraries represent progressively refined, more stringently filtered predicted-BBB-permeable chemical spaces suitable for CNS-focused virtual screening. The numbering used here is applied consistently throughout the manuscript, including the computational case study described below.

### 4.6. Physicochemical, Similarity, Fragment, and Scaffold Profiling

The physicochemical properties of the predicted BBB-permeable molecules were compared against reference sets, including B3DB-positive (B3DB_pos), B3DB-negative (B3DB_neg), DrugBank-approved, and a matched predicted non-permeable set (Pred_BBB_neg) constructed from the Enamine REAL lead-like collection using stringent counter-thresholds (DeepBBB_V1_BC cutoff = 0, DeepBBB_V1_RG cutoff = −1.7, DeepBBB_V2_BC cutoff = 1 × 10^−10^). Six descriptors were summarized: calculated logP, topological polar surface area (TPSA), molecular weight (MW), hydrogen-bond donors (HBD), hydrogen-bond acceptors (HBA), and rotatable bonds (RotB). Chemical diversity was assessed using pairwise and nearest-neighbor Tanimoto similarity on circular (ECFP-type) fingerprints. Fragment composition was summarized as observed fragment-occurrence frequencies across sets, and scaffold structure was summarized using Bemis–Murcko frameworks, including scaffold counts and the fraction of scaffolds not present in B3DB_pos. Where a defined statistical enrichment test with a stated denominator was not available, fragment results are described as frequently represented rather than statistically enriched.

### 4.7. Prospective PAMPA-BBB Assay

The operational feasibility of the DeepBBB workflow was examined prospectively using two PAMPA-BBB campaigns.

In the first campaign, a subset of high-ranking compounds predicted by DeepBBB_V1 (selected from ChemDiv) was evaluated by WuXi AppTec (Shanghai, China) using their standardized PAMPA-BBB protocol. This external assessment enabled rapid evaluation and informed subsequent refinement.

In the second campaign, a larger set of compounds prioritized using DeepBBB_V1_BC/RG together with DeepBBB_V2_BC (applied to the Enamine REAL lead-like library) was tested using an internally implemented PAMPA-BBB protocol based on the established literature methods [13,14]. Dimethyl sulfoxide (DMSO) and dodecane were purchased from Shanghai Aladdin Biochemical Technology Co., Ltd. (Shanghai, China). Porcine brain polar lipids (PBL) were obtained from Avanti Polar Lipids, Inc. (Alabaster, Alabama). Further, 96-well filter plates (polycarbonate membrane, pore size 0.45 µm) and receiver plates were purchased from Corning Inc. (Corning, NY, USA), and 96-well UV plates were obtained from LABSELECT (Beijing, China). Commercial control compounds were supplied by MedChemExpress (Junction, NJ, USA), and test compounds were obtained from Enamine Ltd. (Kyiv, Ukraine). Stock solutions of control drugs and test compounds were prepared in DMSO at 10 mM and diluted with PBS (pH 7.4 ± 0.1) to a working concentration of 80 µM. Donor plates were pre-coated with 6 µL of dodecane-based PBL (20 mg/mL); after 10 min, 150 µL of working solution was added to each donor well, and receiver wells were filled with 300 µL of PBS (pH 7.4). The donor and receiver plates were assembled and incubated in the dark for 6 h. Compound concentrations were determined with a UV microplate spectrophotometer (PerkinElmer VICTOR or Nivo, Turku, Finland) by measuring absorbance in donor, receiver, and control wells, with technical replicates per compound recorded at the assay wavelengths.

Effective permeability coefficients ($P_{e}$, cm·s^−1^) were calculated from standard curves according to Equation (1):

(1) $$P_{e}=-\frac{V_{D}V_{A}}{(V_{D}+V_{A})At}\;\ln\left(1-\frac{C_{A}(t)}{C_{eq}}\right)\;$$

where $V_{D}$ and $V_{A}$ are the donor and acceptor compartment volumes ($V_{D}$ = 0.15 cm^3^, $V_{A}$ = 0.30 cm^3^), A is the membrane surface area (0.28 cm^2^), t is the incubation time (6 h = 21,600 s), $C_{A}(t)$ is the acceptor concentration at time t, and $C_{eq}$ is the equilibrium concentration.

### 4.8. PAMPA-BBB Data Classification and Hit-Fraction Calculation

Following the literature thresholds [13,14], compounds with Pe > 4 × 10^−6^ cm·s^−1^ were classified as PAMPA-BBB-positive (CNS+, above the apparent passive-permeability threshold), compounds with Pe < 2 × 10^−6^ cm·s^−1^ as PAMPA-BBB-negative (CNS−, below the lower threshold), and compounds with intermediate Pe (2–4 × 10^−6^ cm·s^−1^) as borderline (CNS+/CNS−). Compounds for which a reliable Pe value could not be assigned under the assay-specific quantitation and quality-control procedures were recorded as not quantifiable (N/A). N/A was not interpreted as Pe = 0 or as a CNS-negative result. The available summary records do not permit assignment of a single cause to all N/A outcomes. Positive and negative controls were excluded from hit-fraction denominators. The primary reported denominator is all tested candidate compounds (the all-tested molecular-level PAMPA-BBB-positive fraction). Exact (Clopper–Pearson) binomial 95% confidence intervals were computed for these fractions. A quantifiable-only sensitivity fraction is reported in the Supplementary Information.

### 4.9. Computational Downstream Prioritization: Illustrative Case Study

To illustrate how BBB-focused libraries can support target-based CNS prioritization, human glutaminyl cyclase (PDB ID 3PBB) was used as a representative target. Predicted BBB-compatible candidates derived from the Enamine REAL lead-like collection were ranked using the graph-based affinity model Graph_RG. Sequential filtering by Graph_RG score progressively reduced the candidate space to compact panels of top-ranked putative binders suitable for purchase and experimental follow-up. This case study is presented as a computational prioritization illustration; it does not include experimental target-binding measurements and is not described as experimental target validation.

## 5. Conclusions

DeepBBB is best characterized as a graph-based, data-composition-aware screening workflow for constructing BBB-focused virtual libraries and prioritizing compounds for PAMPA-BBB evaluation. Combining data-centric model design with prospective experimental testing, the workflow links ultra-large virtual screening to experimentally testable candidates and identifies PAMPA-BBB-positive compounds in two sequential prospective PAMPA-BBB campaigns (all-tested molecular-level positive fractions of 8.3% and 14.3%). The approach provides a practical strategy for incorporating BBB compatibility as an early constraint in CNS drug discovery, while scaffold-aware retrospective validation, raw-assay reproducibility, chemotype-level interpretation of prospective positives, and experimental target validation remain priorities for future, data-complete work.

## Figures and Tables

**Table 1:** Construction summary of the three BBB-focused specialized libraries.

Library	Source Collection	Model Filters and Thresholds	Retained Compounds	Notes
Specialized Library 1	ChemDiv (~1.5 million)	DeepBBB_V1_BC ≥ 0.99; DeepBBB_V1_RG ≥ 0.3	21,991	Initial BBB-focused ChemDiv subset
Specialized Library 2	Enamine REAL lead-like (~1.7 billion)	DeepBBB_V1_BC = 1.0; DeepBBB_V1_RG ≥ 0.5	4,808,885	Stringent single-model (V1) filter
Specialized Library 3	Enamine REAL lead-like (~1.7 billion)	DeepBBB_V1_BC = 1.0; DeepBBB_V1_RG ≥ 0.5; DeepBBB_V2_BC = 1.0	151,790	Joint V1 + V2; most stringently filtered predicted-BBB-permeable set (Pred_BBB_pos)

Reported library sizes are summary counts from the screening workflow; the underlying ultra-large commercial libraries are third-party resources and are not redistributed. The library numbering above is used consistently in the Results and in the computational case study.

**Table 2:** Second prospective PAMPA-BBB campaign (in-house protocol): permeability of top model-ranked candidate compounds.

Number	Compound ID	Mean Pe (×10^−6^ cm·s^−1^)	Mean % Solution Recovery	Classification ᵃ
Positive control	Metoprolol	7.88	101.58%	CNS+
Negative control	Nadolol	1.91	97.18%	CNS−
1	Z9837060316	0.80	81.31%	CNS−
2	Z7574072689	N/A	91.87%	Not quantifiable
3	Z7574071984	0.26	94.42%	CNS−
4	Z7574066179	1.32	94.41%	CNS−
5	Z9837060346	0.83	95.86%	CNS−
6	Z9837060350	1.90	107.72%	CNS−
7	Z9837060354	N/A	96.55%	Not quantifiable
8	Z9837060372	0.10	93.14%	CNS−
9	Z9837060376	0.25	94.47%	CNS−
10	Z1619878059	N/A	97.94%	Not quantifiable
11	Z1652480351	1.52	108.70%	CNS−
12	Z9006085310	1.83	111.99%	CNS−
13	Z9001604329	N/A	84.87%	Not quantifiable
14	Z9837060424	2.11	98.95%	Borderline (CNS+/CNS−)
15	Z9837060428	0.18	82.77%	CNS−
16	Z8536625717	0.70	109.44%	CNS−
17	Z9837060439	0.35	104.53%	CNS−
18	Z7885935820	3.18	94.43%	Borderline (CNS+/CNS−)
19	Z9837060450	9.41	88.28%	CNS+
20	Z9837060455	N/A	85.27%	Not quantifiable
21	Z9837060457	10.84	92.83%	CNS+
22	Z9837060459	6.88	54.37%	CNS+
23	Z9837060461	10.16	76.32%	CNS+
24	Z9837060464	N/A	87.47%	Not quantifiable
25	Z9837060468	N/A	83.00%	Not quantifiable
26	Z9837060469	3.38	61.66%	Borderline (CNS+/CNS−)
27	Z9837060475	N/A	75.84%	Not quantifiable
28	Z6598975093	N/A	87.90%	Not quantifiable
29	Z4431328388	N/A	101.83%	Not quantifiable
30	Z9837060504	0.05	84.10%	CNS−
31	Z3995438680	N/A	73.27%	Not quantifiable
32	Z4609010601	1.51	108.29%	CNS−
33	Z9823380110	N/A	81.74%	Not quantifiable
34	Z9823380142	N/A	41.71%	Not quantifiable
35	Z8242448685	7.48	102.08%	CNS+

ᵃ Thresholds as in Table 3. Summary of 35 tested candidate compounds: 5 PAMPA-BBB-positive (CNS+), 3 borderline, 13 not quantifiable (N/A), and 14 PAMPA-BBB-negative with quantifiable low Pe. All-tested molecular-level PAMPA-BBB-positive fraction: 5/35 = 14.3% (exact 95% CI 4.8–30.3%); controls excluded from the denominator. N/A entries are not assigned Pe = 0 and are not counted as ordinary CNS-negative measurements.

**Table 3:** First prospective PAMPA-BBB campaign (WuXi AppTec, external protocol): permeability of top model-ranked candidate compounds.

Number	Compound ID	Mean Pe (×10^−6^ cm·s^−1^)	Mean % Solution Recovery	Classification ᵃ
Positive control	Carbamazepine	6.18	103.64%	CNS+
Negative control	Nadolol	0.02	99.95%	CNS−
1	3381-0850	N/A	48.86%	Not quantifiable
2	3381-0695	0.04	8.74%	CNS−
3	V023-3856	N/A	58.68%	Not quantifiable
4	D168-0083	N/A	9.05%	Not quantifiable
5	V010-2950	N/A	58.74%	Not quantifiable
6	3381-0647	N/A	19.33%	Not quantifiable
7	SB95-0969	0.04	8.74%	CNS−
8	FF01-5568	7.23	35.06%	CNS+
9	D399-2452	0.72	2.47%	CNS−
10	V011-3162	N/A	22.31%	Not quantifiable
11	D168-0058	N/A	16.77%	Not quantifiable
12	V023-9426	0.04	79.90%	CNS−

ᵃ Following the literature thresholds, compounds with Pe > 4 × 10^−6^ cm·s^−1^ were classified as PAMPA-BBB-positive (CNS+, above the apparent passive-permeability threshold) and compounds with Pe < 2 × 10^−6^ cm·s^−1^ as PAMPA-BBB-negative (CNS−). Compounds with Pe between 2 and 4 × 10^−6^ cm·s^−1^ were borderline (CNS+/CNS−). N/A = not quantifiable: permeability classification not determined; this is not equivalent to a CNS− measurement. All-tested molecular-level PAMPA-BBB-positive fraction: 1/12 = 8.3% (exact 95% CI 0.2–38.5%); controls excluded from the denominator.

## Data Availability

Model implementations, associated scripts, and usage examples are available in the public GitHub repository (https://github.com/haiping1010/DeepBBB, accessed on 10 August 2026); trained model checkpoints are not included in the repository. The processed summary tables, extracted PAMPA-BBB tables, redrawn figure plot-data, figure scripts, and model-metric summary tables generated for this revision are provided as Supplementary Data accompanying this manuscript. The Enamine REAL lead-like collection, ChemDiv collection, Enamine Hit Locator Library, and DrugBank are third-party resources and cannot be redistributed by the authors. The original sample-level split assignments, per-compound predictions, model checkpoints, random seeds, raw plate-level PAMPA-BBB records, and verified machine-readable structures matching the assay identifiers were not found in the local revision archive and therefore cannot be provided with this revision. A GitHub commit hash corresponding to the original training runs could not be verified from the local archive and is therefore not stated. The original contributions presented in this study are included in the article/Supplementary Materials. Further inquiries can be directed to the corresponding authors.

## Supplementary Materials

The following supporting information can be downloaded at: https://www.mdpi.com/article/10.3390/ph19081319/s1.
