# Supplementary material for: DeepBBB: A Data-Composition-Aware Graph Screening Workflow for BBB-Focused CNS Library Construction and Prospective PAMPA-BBB Evaluation
Source: Pharmaceuticals (Basel). 2026 Aug 21;19(8):1319. doi: 10.3390/ph19081319 (PMC13516556; doi:10.3390/ph19081319)
Supplement: Supplementary file 1 [file pharmaceuticals-19-01319-s001.zip › pharmaceuticals-4441809-supplementary.pdf]

Supplementary Information

DeepBBB: A Data-Composition-Aware Graph Screening Workflow for BBB-Focused CNS Library Construction and Prospective PAMPA-BBB Evaluation

Ziying Xu, Wei Xia, Haiqiang Wu, Haiping Zhang

Supplementary Methods

S1. Evidence boundary and data provenance

This manuscript reassembles a complete workflow from the available materials. To keep every claim aligned with its supporting evidence, data were treated as a hierarchy: per-molecule descriptor/SMILES files (record-level or processed record-level evidence), summary tables (model-metric and PAMPA-BBB summaries), and static figures (workflow schematics and training-curve images). Model-performance values are reported as archived retrospective metrics on the original training/test split; per-sample predictions, exact split assignments, training checkpoints, and random seeds were not re-derived, so receiver-operating-characteristic reconstruction, calibration, scaffold-split evaluation, and train–test leakage auditing were not performed in the present work. PAMPA-BBB results are reported at the level of per-compound mean permeability and categorical assignment; raw plate-level replicate measurements were not re-analyzed, so no replicate-level dispersion statistics are introduced.

S2. PAMPA-BBB classification and denominator rules

Compounds were classified by effective permeability (Pe): PAMPA-BBB-positive (CNS+) for Pe > 4×10^{-6} cm·s^{-1}, PAMPA-BBB-negative (CNS−) for Pe < 2×10^{-6} cm·s^{-1}, and borderline (CNS+/CNS−) for intermediate Pe. Compounds whose permeability could not be reliably quantified were recorded as not quantifiable (N/A), were not assigned Pe = 0, and were not counted as ordinary CNS-negative measurements. Positive and negative controls were excluded from hit-fraction denominators. Exact (Clopper–Pearson) binomial 95% confidence intervals were computed for the all-tested molecular-level PAMPA-BBB-positive fractions.

The all-tested denominator for the second campaign is the 35 candidate compounds listed in Table 2; five were PAMPA-BBB-positive, giving 5/35 = 14.3%. Not-quantifiable compounds were reported separately and were not treated as ordinary CNS-negative measurements. The quantifiable-only sensitivity fractions are reported for transparency and are not used as the primary readout.

Campaign	Tested n	Positive n	Borderline n	N/A n	CNS − n	All-tested fraction	Exact 95% CI	Quantifiable-only fraction
First (external)	12	1	0	7	4	1/12 = 8.3%	0.2–38.5%	1/5 = 20.0%

Campaign	Tested n	Positive n	Borderline n	N/A n	CNS − n	All-tested fraction	Exact 95% CI	Quantifiable-only fraction
Second (in-house)	35	5	3	13	14	5/35 = 14.3%	4.8–30.3%	5/22 = 22.7%

Quantifiable-only fractions are reported for transparency and are not used as the primary readout.

S3. Expanded PAMPA-BBB assay protocol (second campaign, in-house)

Stock solutions of control drugs and test compounds were prepared in DMSO at 10 mM and diluted with PBS (pH 7.4 ± 0.1) to a working concentration of 80 μM. Donor plates (96-well filter plates, polycarbonate membrane, 0.45 μm pore size) were pre-coated with 6 μL of dodecane-based porcine brain polar lipid (PBL, 20 mg/mL). After 10 minutes, 150 μL of working solution was added to each donor well and receiver wells were filled with 300 μL of PBS (pH 7.4). Assembled donor/receiver plates were incubated in the dark for 6 hours. Compound concentrations were measured on a UV microplate spectrophotometer (PerkinElmer VICTOR or Nivo) in donor, receiver, and control wells. Effective permeability (P_e) was calculated from standard curves using the formula given in the main Methods (Equation 1), with donor and acceptor compartment volumes V_D = 0.15 cm^3 and V_A = 0.30 cm^3, membrane area A = 0.28 cm^2, and incubation time t = 21,600 s.

Supplementary Figures

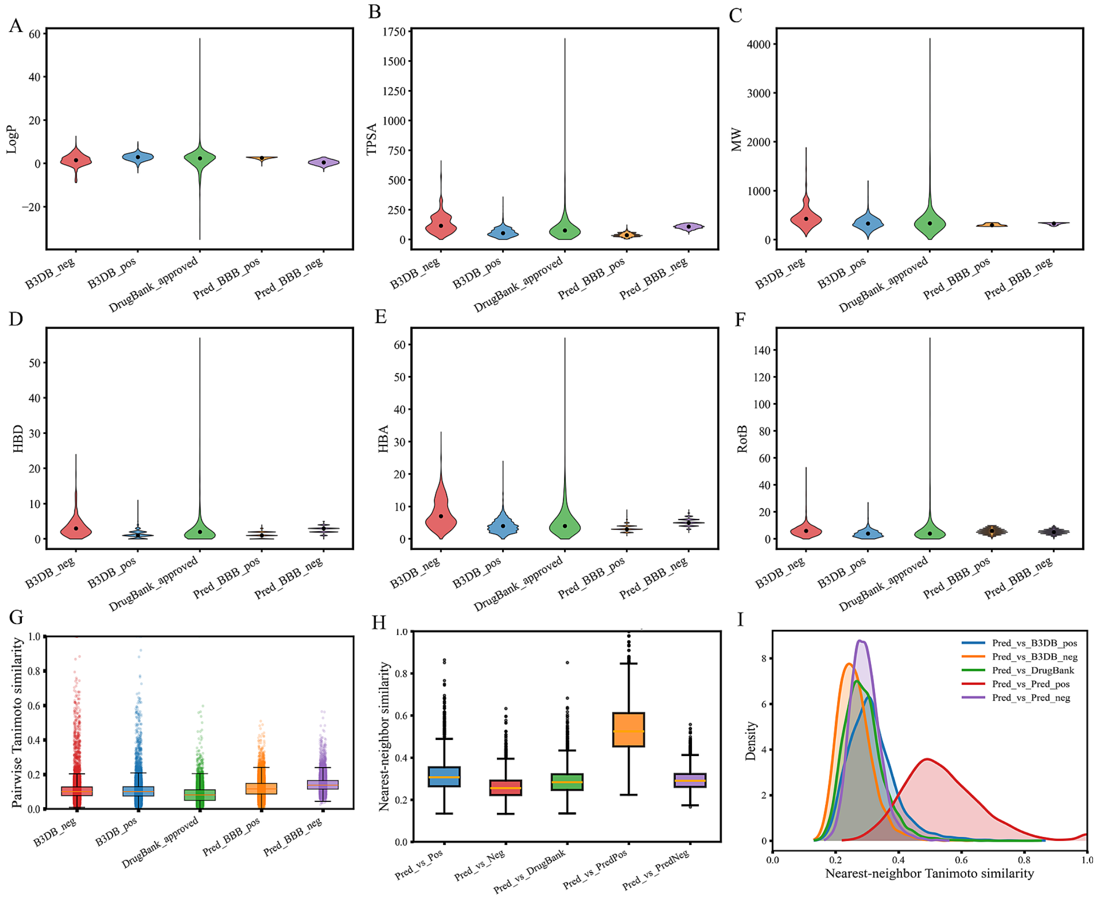

Figure S1. Archived physicochemical and Tanimoto-similarity panels retained as a static figure. The original physicochemical figure, including the pairwise and nearest-neighbor Tanimoto similarity panels (G–I), is retained here because the per-pair similarity matrices were not available for full redrawing. The corresponding descriptor distributions (A–F) are redrawn from processed descriptor tables as main-text Figure 5.

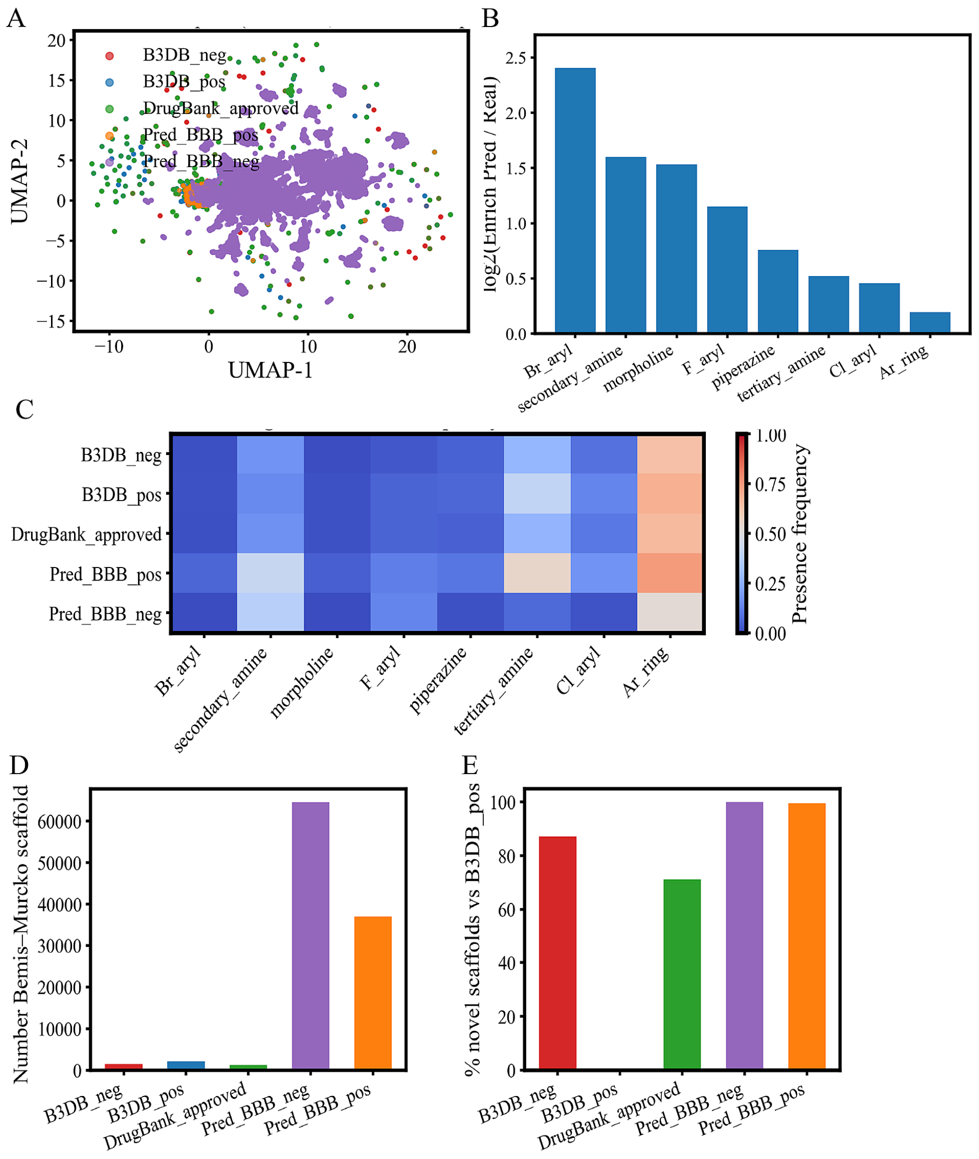

Figure S2. Archived UMAP, fragment-occurrence heat map, and scaffold-novelty panels retained as a static figure. The original fragment/scaffold figure is retained here because the UMAP coordinates and the per-dataset fragment-occurrence heat-map matrix were not available for full redrawing. The redrawn Bemis–Murcko scaffold-count and frequently represented fragment summaries are shown as main-text Figure 6.

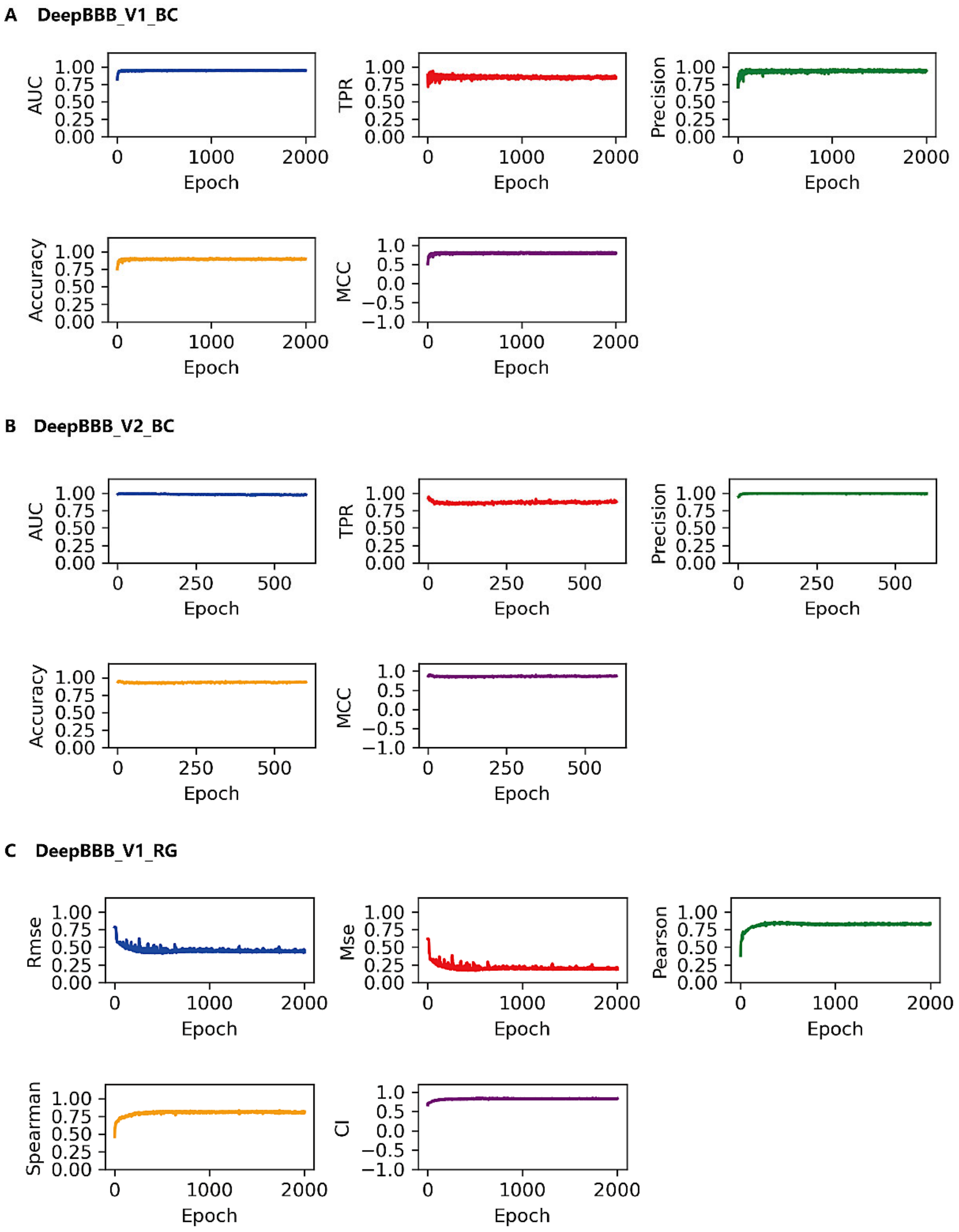

Figure S3. Archived original test-split metric trajectories for DeepBBB_V1_BC, DeepBBB_V2_BC, and DeepBBB_V1_RG. (A) Classification metrics (AUC, TPR, precision, accuracy, MCC) for DeepBBB_V1_BC across archived training epochs. (B) Classification metrics for DeepBBB_V2_BC across archived training epochs. (C) Regression metrics (RMSE, MSE, Pearson, Spearman, CI) for DeepBBB_V1_RG across archived training epochs. These trajectories are retained as static archived figures and are shown descriptively; they are not used here to claim independently optimized model selection.

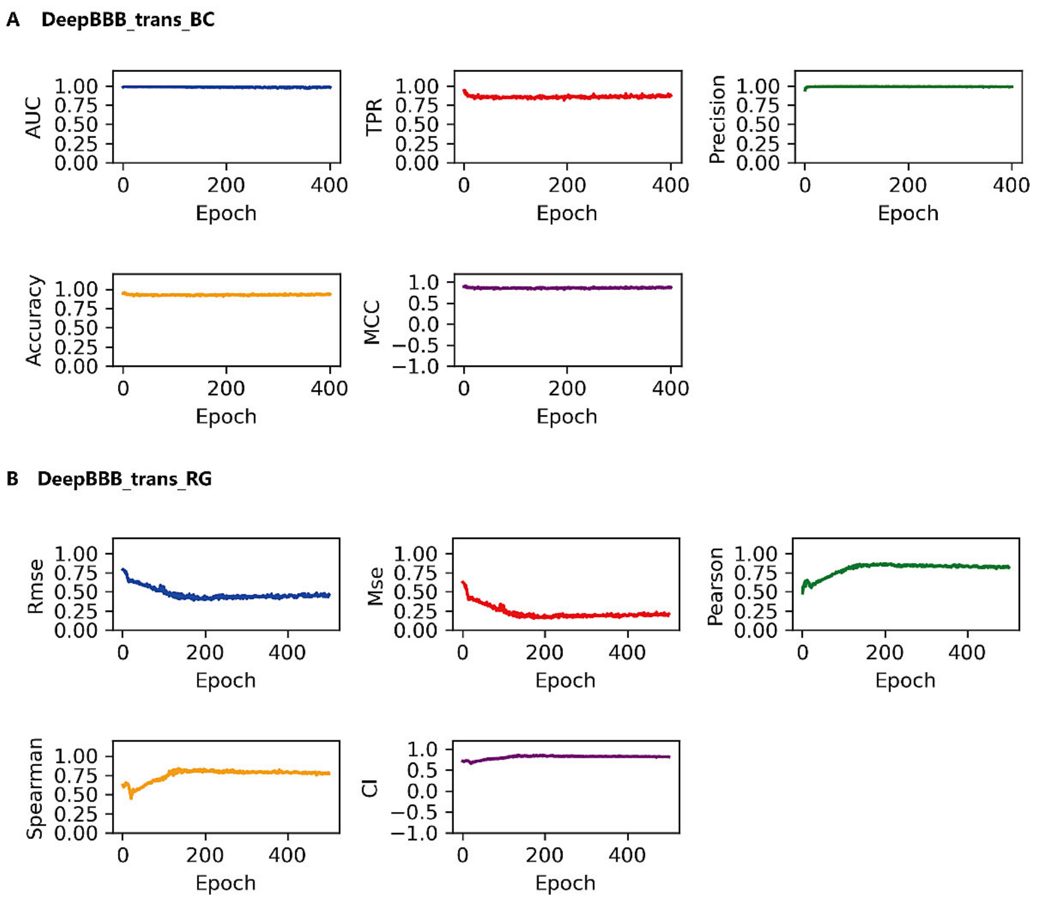

Figure S4. Archived original test-split metric trajectories for DeepBBB_trans_BC and DeepBBB_trans_RG. (A) Classification metrics for DeepBBB_trans_BC across archived training epochs. (B) Regression metrics for DeepBBB_trans_RG across archived training epochs. These trajectories are retained as static archived figures and are shown descriptively; they are not used here to claim independently optimized model selection.

Supplementary Tables

Tables S1–S5. Archived original test-split metrics at the selected application epoch for each DeepBBB model. Values are archived original test-split metrics at the application checkpoint retained for downstream screening. Complete archived epoch-level trajectories for all five models are provided as Supplementary Data File S2. These metrics are shown descriptively and are not used here to claim independently optimized model selection.

Model	Task	Selected epoch	Metric 1	Metric 2	Metric 3
DeepBBB_V1_BC (Table S1)	Classification	2000	AUC 0.95	ACC 0.90	MCC 0.80
DeepBBB_V2_BC (Table S2)	Classification	100	AUC 0.99	ACC 0.93	MCC 0.87
DeepBBB_trans_BC (Table S3)	Classification	100	AUC 0.99	ACC 0.92	MCC 0.85
DeepBBB_V1_RG (Table S4)	Regression	2000	RMSE 0.43	Pearson r 0.84	CI 0.84
DeepBBB_trans_RG (Table S5)	Regression	200	RMSE 0.42	Pearson r 0.85	CI 0.84

Classification metrics: AUC, accuracy (ACC), Matthews correlation coefficient (MCC). Regression metrics: root-mean-square error (RMSE), Pearson correlation coefficient (r), concordance index (CI).

Table S6. Physicochemical descriptor summary by set (means). Mean descriptor values per set; the complete mean/median/SD breakdown for all six descriptors is provided as Supplementary Data File S3.

Set	n	logP	TPSA (Å^2)	MW	HBD	HBA	RotB
B3DB_neg	2851	1.46	133.86	464.63	3.37	7.74	6.25
B3DB_pos	4956	2.87	60.04	339.90	1.23	4.08	3.92
DrugBank_approved	2610	1.71	101.14	392.32	2.39	5.52	5.97
Pred_BBB_neg	128170	0.45	107.01	323.71	2.64	5.16	5.30
Pred_BBB_pos	151790	2.39	38.89	303.21	1.12	3.29	5.74

Table S7A. Fragment-occurrence summary (top 12 by significance rank). Fragment frequencies are observed frequencies in the predicted-positive (Pred_BBB_pos) versus background (Real) sets. The odds-ratio and Benjamini–Hochberg-adjusted p-value (FDR) columns are reproduced from the available fragment-comparison table and are reported descriptively; the main-text language uses "frequently represented" rather than asserting a validated enrichment test. The complete table is provided as Supplementary Data File S5.

Fragment	freq (Pred)	freq (Real)	odds ratio	log2(enrichment)	FDR (BH)
secondary_amine	0.445	0.147	4.66	1.60	0.0
morpholine	0.322	0.119	3.53	1.44	1.4×10^{-237}
sulfonamide	0.0001	0.024	0.005	−7.53	2.3×10^{-158}
urea	0.0023	0.038	0.057	−4.07	9.5×10^{-140}
tertiary_amine	0.595	0.414	2.08	0.52	2.0×10^{-139}
indole	0.0015	0.028	0.051	−4.26	1.9×10^{-109}
pyridine	0.0071	0.045	0.151	−2.67	3.0×10^{-95}
anilide	0.079	0.167	0.429	−1.08	6.3×10^{-87}
Br_aryl	0.071	0.013	5.67	2.42	9.7×10^{-80}
F_aryl	0.137	0.062	2.42	1.15	4.8×10^{-63}
Ar_ring	0.803	0.702	1.73	0.19	1.4×10^{-62}
pyrimidine	0.0075	0.034	0.217	−2.17	3.0×10^{-52}

Table S7B. Bemis–Murcko scaffold summary. Scaffold counts, singleton-scaffold counts, and the fraction of scaffolds not present in B3DB_pos, for each set. This is a library-level processed summary and is not a scaffold-level validation of the prospectively tested compounds. The complete table is provided as Supplementary Data File S5.

Set	n molecules	n scaffolds	singleton scaffolds (%)	scaffolds not in B3DB_pos (%)
B3DB_pos	4956	2239	1604 (71.6%)	0 (0.0%)
DrugBank_approved	2610	1361	1117 (82.1%)	975 (71.6%)
Pred_BBB_pos	151729	38807	26412 (68.1%)	38635 (99.6%)

Table S8. Archived model-prioritized Enamine selection records. Thirty-three Enamine candidates prioritized from Specialized Library 3 are listed with internal Enamine identifiers and DeepBBB scores; the first ten are shown below and the complete 33-row table is provided as Supplementary Data File S1. These archived selection records are not used as the assay denominator. The experimental denominator for the second campaign is

the 35 tested candidate compounds listed in Table 2. A verified mapping between these selection identifiers and the Table 2 catalog (Z…) identifiers was not available in the submitted archive; therefore, Table S8 is provided only as an archived selection-record table.

Enamine ID	DeepBBB_RG	DeepBBB_V1_BC	DeepBBB_V2_BC
s_2230_***678974***_19991558	1.448	1	1
s_2230_***678974***_22391464	1.406	1	1
s_2230_***22405236***_7690762	1.634	1	1
s_2230_***22405236***_8697880	1.416	1	1
s_2230_***22405236***_10121778	1.301	1	1
s_2230_***22406988***_7690762	1.566	1	1
s_2230_***22406988***_8697880	1.406	1	1
s_2230_***25585326***_22994194	1.400	1	1
s_270004_***17786142***_7547488	1.625	1	1
m_2230_***15607912***_8697880	1.483	1	1

(Rows 11–33 are continued in Supplementary Data File S1, the complete machine-readable selection-record table.)

Table S9. Canonical structures and model scores for the 33 archived model-prioritized Enamine selection records. For each of the 33 records already listed in Table S8, the canonical SMILES string was recovered from the archived predicted-library structure file by matching the Enamine synthon-style identifier, and is reported together with the archived DeepBBB_RG, DeepBBB_V1_BC and DeepBBB_V2_BC scores. All 33 identifiers matched exactly. These records are the archived selection records; no mapping between the synthon-style identifiers and the catalogue numbers of the 35 assayed compounds was available in the local archive, so the structures and scores in this table cannot be linked to individual PAMPA-BBB outcomes, and this table is not the assay denominator. Provided in full as Supplementary Data File S6.

No.	Enamine ID	Canonical SMILES	DeepBBB_RG	DeepBBB_V1_BC	DeepBBB_V2_BC
1	s_2230___678974___19991558	CC(C1=CC=C(I)C=C1)N1CCN(C)CC1	1.448	1	1
2	s_2230___678974___22391464	CC(C1=CC=CC(I)=C1)N1CCN(C)CC1	1.406	1	1
3	s_2230___22405236___7690762	CN1CCN(CCN(C)C2CCC3=C2C=CC=C3Cl)CC1	1.634	1	1
4	s_2230___22405236___8697880	CN1CCN(CCN(C)C2CCC3=CC=C(Cl)C=C32)CC1	1.416	1	1
5	s_2230___22405236___10121778	CN1CCN(CCN(C)C2CCC3=CC(Cl)=CC=C32)CC1	1.301	1	1
6	s_2230___22406988___7690762	CN1CCN2CCN(C3CCC4=C3C=CC=C4Cl)CC2C1	1.566	1	1
7	s_2230___22406988___8697880	CN1CCN2CCN(C3CCC4=CC=C(Cl)C=C43)CC2C1	1.406	1	1
8	s_2230___25585326___22994194	CN(C)CCN(C)CCN(C)C1CCC2=C1C=CC=C2Cl	1.4	1	1
9	s_270004___17786142___7547488	CN1CCCN(C2CN(CC3=CC=C(Cl)C(Cl)=C3)C2)CC1	1.625	1	1
10	m_2230___15607912___8697880	CN1CCN(C2CCC3=CC=C(Cl)C=C32)CC1CF	1.483	1	1
11	m_2230___15607912___10121778	CN1CCN(C2CCC3=CC(Cl)=CC=C32)CC1CF	1.362	1	1
12	s_2230___7912950___7690762	CN1CCN(CCCN(C)C2CCC3=C2C=CC=C3Cl)CC1	1.548	1	1
13	s_2230___7912950___8697880	CN1CCN(CCCN(C)C2CCC3=CC=C(Cl)C=C32)CC1	1.39	1	1
14	s_2230___28254758___12297324	CN(CCN1CCCC1)C1CC2=CC=C(Cl)C=C2C1	1.376	1	1
15	s_2230___678974___14479670	CN1CCN(C2CCC3=CC=C(Br)C=C32)CC1	1.303	1	1
16	s_2230___21228944___19991558	CC(C1=CC=C(I)C=C1)N(C)C1CCN(C)C1	1.394	1	1
17	s_2230___21228944___22391464	CC(C1=CC=CC(I)=C1)N(C)C1CCN(C)C1	1.338	1	1
18	s_270004___24163444___24721108	COC(CN(C)CC(OC)C1=CC=C(Cl)C=C1)OC	1.554	1	1
19	s_270004___24171156___24714688	CN1CC2CC(C1)N2CC1=CC(C(F)(F)Cl)=CC=C1	1.326	1	1
20	s_270004___24155796___24594634	CN(C)C1CN(CCCC2=CC=C(I)C=C2)C1	1.383	1	1

No.	Enamine ID	Canonical SMILES	DeepBBB_RG	DeepBBB_V1_BC	DeepBBB_V2_BC
21	m_270004___24159022___14479272	CN1CC(N2CCN(CC3=CC=CC4=CC(Cl)=CC=C34)CC2)C1	1.494	1	1
22	s_270004___24159952___7548744	CN1CCN(C2CN(CC3=CC=C(Cl)C(C(F)(F)F)=C3)C2)CC1	1.471	1	1
23	s_270004___24159022___24714858	CN1CC(N2CCN(CC3=CC=CC4=CC=C(Cl)C=C34)CC2)C1	1.467	1	1
24	m_270004___24159952___14479272	CN1CCN(C2CN(CC3=CC=CC4=CC(Cl)=CC=C34)C2)CC1	1.463	1	1
25	s_270004___24159952___24714858	CN1CCN(C2CN(CC3=CC=CC4=CC=C(Cl)C=C34)C2)CC1	1.448	1	1
26	s_270004___24159952___8127734	CN1CCN(C2CN(CC3=CC(C(F)(F)F)=CC=C3Cl)C2)CC1	1.407	1	1
27	s_270004___24159952___9105414	CN1CCN(C2CN(CC3=CC(C(F)(F)F)=CC(Cl)=C3)C2)CC1	1.347	1	1
28	s_270004___24164224___24714858	CN1CCN2CCN(CC3=CC=CC4=CC=C(Cl)C=C34)CC2C1	1.424	1	1
29	s_2230___22406988___6548402	CN1CCN2CCN(CCCC3=CC=C(C(F)(F)F)C=C3)CC2C1	1.447	1	1
30	s_269982___7368798___16619354	CNC1CCCN(CC2CCC3=CC=CC=C3CC2)C1	1.358	1	1
31	s_270004___24159952___7547488	CN1CCN(C2CN(CC3=CC=C(Cl)C(Cl)=C3)C2)CC1	1.655	1	1
32	s_270004___24164224___7547488	CN1CCN2CCN(CC3=CC=C(Cl)C(Cl)=C3)CC2C1	1.674	1	1
33	m_271302___22976938___25549918	CNC1CCCN(CC2CCC3=CC=CC=C3C2)CC1	1.407	1	1

Supplementary Data

The following machine-readable files accompany this manuscript as Supplementary Data:

- **Supplementary Data File S1 —** complete 33-row Enamine selection-record table (Table S8). File name: Supplementary_Data_File_S1_Table_S1_selection_records.xlsx
- **Supplementary Data File S2 —** complete archived epoch-level model metrics for all five models (Tables S1–S5). File name: Supplementary_Data_File_S2_Tables_S2_to_S6_model_metrics.xlsx
- **Supplementary Data File S3 —** full descriptor summary with mean/median/SD for all sets (Table S6). File name: Supplementary_Data_File_S3_Table_S7_descriptor_summary.xlsx
- **Supplementary Data File S4 —** PAMPA-BBB denominator summary for both campaigns (Supplementary Methods S2). File name: Supplementary_Data_File_S4_pampa_denominator_summary.xlsx
- **Supplementary Data File S5 —** fragment-occurrence and Bemis–Murcko scaffold summaries (Tables S7A and S7B). File name: Supplementary_Data_File_S5_Tables_S8A_S8B_fragment_scaffold_summary.xlsx
- **Supplementary Data File S6 — canonical structures and model scores for the 33 archived Enamine selection records (Table S9). File name: Supplementary_Data_File_S6_Table_S9_selection_records_structures_and_scores.xlsx**
- Redrawn figure plot-data and figure scripts for main-text Figures 4–7, and extracted PAMPA-BBB campaign tables.

A file-by-file listing with descriptions, source, completeness, and redistribution status is provided in the Supplementary Data manifest (file: Supplementary_Data_Manifest_v3.xlsx, also supplied as data_manifest.csv).
